# Opioid-sparing effect of cannabinoids for analgesia: an updated systematic review and meta-analysis of preclinical and clinical studies

**DOI:** 10.1038/s41386-022-01322-4

**Published:** 2022-04-22

**Authors:** Suzanne Nielsen, Louisa Picco, Bridin Murnion, Bryony Winters, Justin Matheson, Myfanwy Graham, Gabrielle Campbell, Laila Parvaresh, Kok-Eng Khor, Brigid Betz-Stablein, Michael Farrell, Nicholas Lintzeris, Bernard Le Foll

**Affiliations:** 1 Monash Addiction Research Centre, Eastern Health Clinical School, Monash University, Frankston, VIC Australia; 2 National Drug and Alcohol Research Centre, UNSW, Sydney, NSW Australia; 3 Discipline of Addiction Medicine, Faculty of Medicine and Health, University of Sydney, Sydney, NSW Australia; 4 Pain Management Research Institute, University of Sydney and Royal North Shore Hospital, Sydney, NSW Australia; 5 Translational Addiction Research Laboratory, Campbell Family Mental Health Research Institute, Centre for Addiction and Mental Health (CAMH), Toronto, ON Canada; 6 The Australian Centre for Cannabinoid Clinical and Research Excellence (ACRE), Newcastle, NSW Australia; 7 Centre Centre for Drug Repurposing and Medicines Research, School of Medicine and Public Health, The University of Newcastle, Newcastle, NSW Australia; 8 School of Health and Behavioural Sciences, University of the Sunshine Coast, Maroochydore, QLD Australia; 9 Drug and Alcohol Services, South Eastern Sydney Local Health District, Surry Hills, NSW Australia; 10 School of Public Health, Faculty of Medicine and Health, University of Sydney, Sydney, NSW Australia; 11 Medicine and Health, UNSW, Kensington, NSW Australia; 12 Department of Pain Management, Prince of Wales Hospital, Randwick, NSW Australia; 13 The University of Queensland Diamantina Institute, The University of Queensland, Dermatology Research Centre, Brisbane, QLD Australia; 14 Departments of Pharmacology and Toxicology, Psychiatry, Family and Community Medicine, University of Toronto, Toronto, ON Canada

**Keywords:** Preclinical research, Translational research

## Abstract

Cannabinoid co-administration may enable reduced opioid doses for analgesia. This updated systematic review on the opioid-sparing effects of cannabinoids considered preclinical and clinical studies where the outcome was analgesia or opioid dose requirements. We searched Scopus, Cochrane Central Registry of Controlled Trials, Medline, and Embase (2016 onwards). Ninety-two studies met the search criteria including 15 ongoing trials. Meta-analysis of seven preclinical studies found the median effective dose (ED_50_) of morphine administered with delta-9-tetrahydrocannabinol was 3.5 times lower (95% CI 2.04, 6.03) than the ED_50_ of morphine alone. Six preclinical studies found no evidence of increased opioid abuse liability with cannabinoid administration. Of five healthy-volunteer experimental pain studies, two found increased pain, two found decreased pain and one found reduced pain bothersomeness with cannabinoid administration; three demonstrated that cannabinoid co-administration may increase opioid abuse liability. Three randomized controlled trials (RCTs) found no evidence of opioid-sparing effects of cannabinoids in acute pain. Meta-analysis of four RCTs in patients with cancer pain found no effect of cannabinoid administration on opioid dose (mean difference −3.8 mg, 95% CI −10.97, 3.37) or percentage change in pain scores (mean difference 1.84, 95% CI −2.05, 5.72); five studies found more adverse events with cannabinoids compared with placebo (risk ratio 1.13, 95% CI 1.03, 1.24). Of five controlled chronic non-cancer pain trials; one low-quality study with no control arm, and one single-dose study reported reduced pain scores with cannabinoids. Three RCTs found no treatment effect of dronabinol. Meta-analyses of observational studies found 39% reported opioid cessation (95% CI 0.15, 0.64, *I*^2^ 95.5%, eight studies), and 85% reported reduction (95% CI 0.64, 0.99, *I*^2^ 92.8%, seven studies). In summary, preclinical and observational studies demonstrate the potential opioid-sparing effects of cannabinoids in the context of analgesia, in contrast to higher-quality RCTs that did not provide evidence of opioid-sparing effects.

## Introduction

Opioids are widely prescribed for chronic pain, but due to concerns related to harms, recommendations have been made to reduce reliance on higher doses [1]. One strategy to reduce opioid dose requirements has been through use of opioid-sparing medicines. Opioid-sparing medicines can (1) delay or prevent the initiation of treatment with opioid analgesics (2) decrease the duration of opioid treatment (3) reduce the total dosages of opioid used or (4) reduce opioid-related adverse outcomes, without causing an unacceptable increase in pain [2].

There is substantial interest in the opioid-sparing potential of cannabinoids in the context of pain management. Preclinical data have consistently demonstrated opioid-sparing effects [3]. Interest from policy makers has been further driven by ecological and epidemiological research [4]; however, highly publicized findings have recently been questioned [5].

The overlapping neuroanatomical distribution of opioid and cannabinoid receptors in the central and peripheral nervous system in areas involved with anti-nociception support potential opioid-sparing effects. Opioids and cannabinoids have comparable neurobiological properties with significant degree of functional interaction [6]. Opioid and cannabinoid receptors are G_i/o_-protein-coupled receptors with similar intracellular signaling mechanisms, including: inhibition of the adenylate cyclase activity, reduced activity of voltage-dependent calcium channels, activation of inwardly-rectifying potassium channels, and stimulation of the MAP kinase cascade. Cannabinoid type-1 (CB1) and mu receptors can interact directly as functional heterodimers when co-expressed in the same neuron [7] and cannabinoid administration may stimulate the synthesis and release of endogenous opioid peptides centrally and peripherally [8]. Each of these properties would predict a synergistic interaction between opioids and cannabinoids, yet further complexity is afforded by the pharmacological profile of the drug. For example, in the case of protean agonists the level of activation of cannabinoid receptors (both constitutive and stimulated) impacts upon the observed pharmacological effect [9, 10], whilst partial agonists such as the endocannabinoid anandamide could act as an antagonist in the presence of a more efficacious agonist [11].

Our previous systematic review and meta-analysis found robust preclinical evidence supporting the opioid-sparing potential of delta-9-tetrahydrocannabinol (THC), but limited clinical research testing the opioid-sparing effects of cannabinoids [3]. With the proliferation of research in the past five years, this review aims to provide an updated synthesis of preclinical and clinical studies on the opioid-sparing effects of cannabinoids.

## Materials and methods

### Search

We conducted an updated systematic literature search in accordance with the Preferred Reporting Items for Systematic Reviews and Meta-Analyses (PRISMA) recommendations [12]. The initial searches conducted on October 29, 2015, had no date limits and the findings have been reported earlier, along with the methods (in lieu of a published/registered protocol) [3]. The updated searches were conducted on December 20, 2020 within Scopus, Cochrane Central Registry of Controlled Trials, Medline, and Embase databases and results were combined with the earlier search. A combination of search terms relating to opioids (e.g., analgesics, opioid*, opiate), cannabinoids (e.g., cannabis, sativex, nabiximol, cannabidiol, tetrahydrocannabinol) and outcomes of interest (e.g., pain, opioid sparing, opioid dose, antinociceptive) were used, consistent with the initial search (Appendix 1). Additional targeted searches of reference lists from identified studies and review articles were conducted to find additional studies not identified by the main searches.

### Study eligibility

Eligible studies included: (i) human or animal studies; (ii) for human studies, controlled clinical and preclinical studies where cannabinoids were administered within a medical or clinical therapeutic framework and the study outlined details of cannabinoid administration; (iii) documented concurrent administration of opioids and cannabinoids; (iv) an outcome of either pain/analgesia (including acute, chronic, cancer and non-cancer and experimental pain studies) or opioid requirements/opioid-sparing.

Studies were excluded based on the following criteria: (i) wrong intervention (e.g., cannabinoid use not defined, no cannabinoid administered, non-concurrent opioid and cannabinoid use, non-therapeutic opioid use); (ii) wrong study design (e.g., case reports, epidemiological studies, reviews, letters without empirical data, commentary or news article); (iii) no outcome measure of interest (i.e., pain/analgesia or opioid dose); (iv) full text unavailable; (v) duplicate manuscript; (vi) abstract where full paper published; (vii) unable to confirm eligibility details, or access required data from authors (Appendix 2).

Titles and abstracts, and full texts were screened independently by two authors (SN, LMP, JM, BM, GC, MG, LP and K-EK) using Covidence software [13]. Where inconsistencies were identified, the authors were able to reach consensus on each occasion.

### Data extraction and outcomes

The same data extraction forms used in the initial review were used. All data were extracted by one of the authors (SN, LMP and BW, BM) and checked by a second author (SN, LP, BM, JM, MG or K-EK). These same authors reviewed and resolved any inconsistencies. For abstracts without a full text, and missing data, attempts were made to contact authors for additional information.

### Outcome measures

For preclinical studies, the primary outcome was the dose of opioid required to give an equivalent antinociceptive effect in the presence and absence of cannabinoids.

### Analysis

#### Preclinical studies

Data were extracted and, where studies that were sufficiently similar in design and outcome measures, meta-analysis was undertaken. For the residual studies, a narrative review was conducted.

To prepare the data for the meta-analysis, the ED_50_ and either confidence limits or standard error were extracted from the relevant literature. ED_50_ is calculated on the log_10_ scale. Therefore, to meet the assumption of normality, the $\log _{10}\;\widehat {ED}_{50}$ must be used in the meta-analysis. The log_10_ of the confidence limits must also be determined to calculate the standard deviation (SD) of the $\log _{10}\;\widehat {ED}_{50}$:

$$SD\left( {{{{{{{{\mathrm{log}}}}}}}}_{10}\;\widehat {ED}_{50}} \right) = \frac{{{{{{{{{\mathrm{log}}}}}}}}_{10}UL - {{{{{{{\mathrm{log}}}}}}}}_{10}\;\widehat {ED}_{50}}}{{1.96}}$$

where UL is the upper confidence limit.

When only standard error was reported, the confidence limits were calculated using the method of Litchfield and Wilcoxon [14] and the above procedure was repeated to calculate the standard deviation. This method also allowed for the inclusion of studies that did not report exact sample sizes for all treatment groups, as sample size was not required for the calculation of standard deviation.

Data for the meta-analysis were analyzed using Review Manager 5.4 (Cochrane Collaboration, Oxford, UK). When calculating the continuous outcome of an equally effective opioid dose (e.g., the log_10_ED_50_ for morphine when administered alone versus when administered with a cannabinoid), the inverse variance statistical method and random effects model were used to compensate for study heterogeneity.

No statistical difference was found in outcomes between the studies that used different rodent species or nociceptive assays. Therefore, the mean difference of ${{{{{\rm{log}}}}}}_{10}ED_{50}$ and the corresponding 95% confidence intervals were calculated. Due to the nature of log calculations, the mean difference—when back-transformed to the original units—represents the response ratio. For easier interpretation, we present the reciprocal of the response rate.

#### Clinical studies

The outcomes of interest in clinical studies were: (1) reduction in total opioid doses, (2) reductions in pain through the addition of a cannabinoid, (3) adverse events, and (4) evidence of abuse liability. A broad range of study designs were considered. Where studies used sufficiently similar methods and outcome measures, meta-analyses were conducted.

#### Clinical trials

Meta-analysis for clinical trials was conducted with Revman 5.4, where medians and interquartile ranges were required to be converted into means and standard deviations to allow inclusion in meta-analyses, we used methods established by Luo et al. [15] and Wan et al. [16].

### Observational studies

For observational studies, meta-analyses on proportions reporting changes in opioid dose outcomes were conducted using a random effect model in Stata (metaprop, code available on request). A pooled prevalence was calculated with 95% confidence intervals for each of the identified outcomes that were comparable; (i) reduced opioid use, (ii) ceased opioid use. For remaining outcomes, a narrative synthesis was conducted.

Clinical studies were scored for quality using the Grading of Recommendations Assessment, Development and Evaluation (GRADE) criteria [17]. Quality ratings were not applied to preclinical studies. As all meta-analyses had less than ten studies funnel plots were not used to assess bias [18].

## Results

Ninety eligible publications representing data from 92 studies were identified; 29 in the initial searches and 63 in the updated searches. Forty preclinical (21 since 2016) and 37 clinical studies (controlled trials *n* = 20 [12 since 2016] and observational *n* = 17 [13 since 2016]) were identified for inclusion (see Appendix 3). Fifteen registered clinical trials, where data were not yet available were also identified.

### Summary of preclinical studies

Forty preclinical studies were identified in which the analgesic effect of opioid and cannabinoid co-administration was examined [19–58]. Sixteen of these studies examined delta-9-THC, while smaller numbers of studies examined 20 other cannabinoids, including agonists mixed CB1/CB2 agonists (CP55,940, WIN55,212-2, HU-210), CB1 agonists (ACEA, ACPA), CB2 agonists (beta-caryophyllene, JWH-015, JWH-133, LY2828360), antagonists/inverse agonists at the CB1 (AM-251) and CB2 receptor (JTE-907) and other cannabinoids (AM1241, cannabinol, cannabidiol [CBD], CP 56,667, delta-8-THC, 11-hydroxy-delta-9-THC, dextronantradol, levonantradol and GP1a) (Table 1 and Appendix 4). Opioids examined included morphine, codeine, and other agonists at the mu, delta or kappa opioid receptor including buprenorphine, etorphine, fentanyl, heroin, oxycodone, hydromorphone, methadone, LAAM, meperidine, pentazocine, spiradoline, tramadol, and SNC80. Most studies used rodents; however, three used rhesus monkeys and one used guinea pigs. The most common antinociceptive assays were of thermal nociception although assays of mechanical and chemical nociception were also utilized.

**Table 1:** Summary of opioid-sparing outcomes in preclinical studies by cannabinoid type.

Cannabinoid type	Potential synergism/opioid-sparing effects	Opioid-sparing effect not clearly observed^a^ or tested
*Mixed CB1/CB2 agonists*		
CP55,940 (mixed CB1/CB2 agonist)	Evidence of opioid-sparing effect: Alsalem et al. 2019 (morphine “potential synergy” mechanical nociception) Maguire and France 2018 (morphine, thermal nociception); Maguire 2013 (Rhesus monkey, morphine, thermal nociception)	Evidence of synergy/opioid-sparing not found: Alsalem et al. 2019 (tramadol, mechanical nociception) Welch 1992 (morphine, thermal nociception); Maguire and France 2016 (spiradoline, thermal nociception) Maguire and France 2018 (etorphine, thermal nociception); Minervini 2017 (spiradoline, thermal nociception)
Delta-9-THC (partial agonist CB1/CB2)	Evidence of opioid-sparing effect: Cox 2007 (morphine, mechanical nociception) Cichewicz 2005 (guinea pigs, fentanyl and buprenorphine, mechanical nociception) Maguire and France 2018 (morphine, thermal nociception) Nguyen 2019 (oxycodone “possibly synergistic, thermal nociception) Nilges 2020 (Rhesus monkeys, heroin, thermal nociception)) Cichewicz 1999 (range of opioid agonists, thermal nociception) Cichewicz 2003 (morphine and codeine, thermal nociception) Li 2008 (Rhesus monkey, morphine, thermal nociception)) Pugh 1996 (morphine, thermal nociception) Smith 1998 (morphine, thermal nociception) Smith 2007(morphine, thermal nociception) Welch 1992(morphine, thermal nociception) Williams 2006 (codeine and morphine, thermal nociception) Williams 2008 (morphine, thermal nociception)	Evidence of synergy/opioid-sparing not found: Maguire and France 2018 (etorphine, thermal nociception) Opioid-sparing/synergism not directly tested: Wakley 2011—synergism not tested, (mechanical nociception) Reche 1996—only one dose of morphine examined (thermal nociception)
HU-210 (mixed CB1/CB2 agonist)	Evidence of potential opioid-sparing effect: Sierra 2019 (SNC80 [delta opioid agonist] mechanical nociception with neuropathic pain model)	Evidence of synergy/opioid-sparing not found: Alsalem et al. 2020 morphine and tramadol, mechanical nociception) Wilson 2008 (morphine, thermal nociception)
WIN55,212–2 (mixed CB1/CB2 agonist)	Evidence of opioid-sparing effect: Alsalem et al. 2020 (tramadol mechanical nociception); Chen et al. 2019 (morphine, thermal nociception and formalin) Yesilurt 2003 (morphine, thermal nociception)	Evidence of synergy/opioid-sparing not found: Alsalem et al. 2020 (not morphine, mechanical nociception)
*CB1 selective agonist*		
ACEA		Evidence of synergy/opioid-sparing not found: Altun 2015 (morphine, thermal nociception)
ACPA		Evidence of synergy/opioid-sparing not found: Auh et al. 2016 (DAMGO, mechanical nociception)
*CB1 antagonist/inverse agonist*		
AM-251 (also has agonist activity at GPR55)		Evidence of synergy/opioid-sparing not found: Altun 2015 (morphine, thermal nociception)
*CB2 selective agonist*		
JWH-015	Evidence of opioid-sparing effect: Grenald et al. 2017 (morphine, mechanical and thermal nociception, formalin pain assay)	Evidence of synergy/opioid-sparing not found: Altun 2015 (morphine, thermal nociception)
Beta-caryophyllene		Evidence of synergy/opioid-sparing not found: Katsuyama 2013 (morphine, capsaicin pain assay)
JWH-133		Evidence of synergy/opioid-sparing not found: Yuill 2017 (morphine, noxious stimuli)
LY2828360	Evidence of opioid-sparing effect: Iyer 2020 (morphine, mechanical nociception)	
*CB2 complex*		
AM1241 (Protean agonist CB2)	Evidence of potential synergy: Zhang 2018 (morphine, thermal and mechanical nociception)	Evidence of synergy/opioid-sparing not found: Stachtari 2016 (tramadol, thermal nociception) Zhang 2017 (morphine, thermal nociception); Zhang 2016 (morphine, thermal and mechanical nociception)
GP1a (CB2 agonist/inverse agonist)	Evidence of potential opioid-sparing effect: Chen et al. 2019 (morphine, thermal nociception)	Evidence of synergy/opioid-sparing not found: Chen et al. 2019 (morphine, formalin pain assay)
*CB2 antagonist*		
JTE-907 (CB2 antagonist)		Evidence of synergy/opioid-sparing not found: Altun 2015 (morphine, thermal nociception)
*Complex*		
CBD (inverse agonist/NAM CB1, partial agonist CB2)		Evidence of synergy/opioid-sparing not found: Neelakantan 2015 (morphine, thermal and chemical nociception)
Cannabinol (partial CB1 inverse agonist or agonist at CB2)		Evidence of synergy/opioid-sparing not found: Nilges 2020 (heroin, thermal nociception)
*Less characterized cannabinoids*		
CP 56,667		Evidence of synergy/opioid-sparing not found: Welch 1992 (morphine, thermal nociception)
Delta-8-THC	Evidence of opioid-sparing effect: Welch 1992 (morphine, thermal nociception)	
11-hydroxy-delta-9-THC	Evidence of opioid-sparing effect: Welch 1992 (morphine, thermal nociception)	
Dextronantradol		Evidence of synergy/opioid-sparing not found: Welch 1992 (morphine, thermal nociception)
Levonantradol	Evidence of opioid-sparing effect: Welch 1992 (morphine, thermal nociception]	

Species were rodents unless otherwise specified; full study details provided in Supplementary material (see Appendix 3).

^a^Where opioid-sparing effect reported as not observed, results were additive rather than synergistic; or no increased analgesic effect was observed.

Evidence of opioid-sparing effects or synergism were found for all mixed CB1/CB2 agonists (CP55,940, delta-9-THC, HU-210, WIN55,212–2). Morphine-induced analgesia increased with the CB1 selective agonist ACEA, though the effect was additive as opposed to synergistic [40]. In contrast, the CB1 selective agonist ACPA, and DAMGO (selective mu agonist) appeared to act antagonistically when administered together in a model of mechanical hyperalgesia [41]. The CB1 antagonist/inverse agonist AM-251 reduced the analgesic effect of morphine [40]. Conflicting outcomes were seen for CB2 selective agonists (some evidence of opioid-sparing effects for GP1a, JWH-015, LY2828360, but not for beta-caryophyllene or JWH-133). JTE-907 (a CB2 antagonist) and cannabinoids with more complex pharmacology (CBD and cannabinol) did not demonstrate opioid-sparing effects. Three less well characterized phytocannabinoids, including delta-9-THC metabolites, also showed evidence of synergy or opioid-sparing effects (delta-8-THC, 11-hydroxy-delta-9-THC and levonantradol), while no opioid-sparing effects were seen for other less well characterized cannabinoids (CP, 56,667 and dextronantradol).

#### Measures of abuse liability

Six studies reported on measures of abuse liability including intracranial self-stimulation (ICSS) [38], conditioned place preference [43, 44], oxycodone self-administration [50], and drug discrimination [32, 33]. None provided evidence that cannabinoids increased abuse liability. CP55,940 had no effect on ICSS with morphine or tramadol [38], JWH105 when co-administered with morphine reduced conditioned place preference, and LY2828360 when administered with morphine blocked condition place preference [43, 44]. THC reduced oxycodone self-administration [50], and attenuated the discriminative stimulus effect of morphine and heroin in nondependent monkeys, but not in dependent monkeys [33]. CP55,940 and WIN55,212 reduced the discriminative stimulus effect of morphine and decreased heroin self-administration, both effects were reversed by the CB1 receptor inverse agonist rimonabant [32].

#### Meta-analysis of preclinical studies

**Fig. 1:**
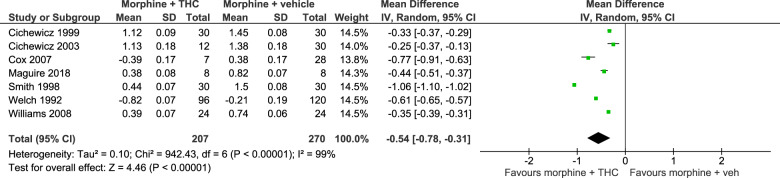
Forrest plot for meta-analysis examining the opioid-sparing effect of delta-9-THC when co-administered with morphine. Note mean difference and standard deviation values are of log_10_*ED*_50_.

Seven studies used sufficiently similar approaches to enable a meta-analysis [19–24, 47] (Fig. 1). All studies included in the meta-analysis used rodents and reported comparable antinociceptive doses of morphine alone and morphine co-administered with delta-9-THC.

Meta-analysis identified an opioid-sparing effect with morphine and delta-9-THC co-administration with one study [47] added to the previous meta-analysis, *Z* = 4.46, *p* < 0.001 (mean difference in log_10_ED_50_ = –0.54 [–0.78, –0.31]). As there was significant heterogeneity in the data (*I*^2^ = 99%), a random effects model was used. When back-transformed to the original units, the response ratio was 3.5 (95% CI 2.04, 6.03) indicating that the median effective dose (ED_50_) of morphine was 3.5 times lower when administered with delta-9-THC compared to when administered alone.

### Results from clinical studies

Thirty-five eligible publications representing 37 clinical studies with 5180 participants provided data relevant to the research question (Table 2).

**Table 2:** Clinical studies.

Study reference	Study design	Population	Observation period	Opioid used	Cannabinoid Used	Comparator	Effect of cannabinoid on opioid dose	Outcome on analgesia observed	GRADE evidence rating and other notes
*a. Experimental pain studies*									
Babalonis 2019	Within-subject crossover, randomized, double-blind placebo-controlled design. Analgesia assessed with cold pressor, pressure algometer, hot thermode, cold hyperalgesia	Healthy volunteers (n=10), aged 18–50 years, without acute or chronic pain conditions and no recent opioid or cannabinoid use. Six female, mean age of 26.3 years	Nine outpatient experimental sessions (8.5 h/session) with a minimum of 48 h separating each session, dronabinol administered 1 h before oxycodone, pain measures up to 6 h after dronabinol administration	Oxycodone 0, 5, 10 mg (oral)	Dronabinol l 0, 2.5, 5 mg (oral)	Placebo dronabinol capsules and placebo oxycodone tablets	Cold pressor test: 2.5 mg dronabinol + 5 mg oxycodone decreased tolerance (17.9 [±2.4] s), compared with the 5 mg oxycodone dose alone (34.3 [±17.7] s)	Pressure algometer: Dronabinol a 2.5 mg dose decreased the analgesic effects of 10 mg oxycodone (no effect from 5 mg dronabinol). No effect on other pain measures (pressure algometer, cold pressor test and hot thermode)	GRADE rating “moderate”, placebo-controlled blinded study, indirect evidence as use of experimental pain. Dronabinol increased abuse liability ratings of oxycodone
Cooper 2018	Within-subject, randomized, placebo-controlled, double-blind study Analgesia was assessed with cold pressor test	Healthy volunteers (*n* = 18) 21–45 years, with who current cannabis use. Six (33%) female, mean age 29.9 years	6 outpatient experimental sessions. Placebo or oxycodone was administered 45 min before cannabis. Observations for 5 h after cannabis administration; repeated pain assessments until 3 h. 72 h washout between sessions	Oxycodone 0, 2.5 or 5.0 mg (oral)	Cannabis cigarettes (0.0 or 5.6% THC content); Participants smoked 70% of an 800 mg cannabis cigarette (CBD content not stated)	Placebo cannabis cigarettes (0% THC); Placebo oxycodone capsules	Cannabis and low dose of oxycodone (2.5 mg) did not elicit analgesia on their own; when administered together, pain (with cold pressor test) was significantly reduced, pointing to the opioid-sparing effects	Cannabis and 2.5 mg and 5 mg oxycodone increased pain threshold on cold pressor test compared to the cannabis alone (*p* < 0.05). Mean reductions from pain (McGill Pain Questionnaire) Placebo 2.2 ± 0.5; THC alone 1.5 ± 0.5; 2.5 mg OXY 2.0 ± 0.5; 2.5 mg OXY + THC; 0.7 ± 0.6; 5 mg OXY 1.7 ± 0.4; 5 mg OXY + THC 1.2 ± 0.4. Pain Intensity and bothersomeness Scales did not differ between cannabis, oxycodone, the combination or placebo	GRADE rating “moderate”, placebo-controlled blinded study, indirect evidence as use of experimental pain Smoked cannabis increased subjective abuse liability measures for oxycodone
Dunn 2021	Double-blind, within-subject randomized, placebo-controlled, human laboratory study using quantitative sensory testing measures of acute (thermal, pressure pain; thermal, punctate probe temporal summation; cold pressor; conditioned pain modulation) and chronic pain (capsaicin 10% topical cream with thermal rekindling)	Healthy adults (*n* = 29) with no history of drug use disorder, 52% female, mean 30.4 years	Five outpatient laboratory sessions (min. 7 days apart). Sessions lasted 8 h. Study drugs co-administered at with hourly pain assessments for 4 h	Hydromorphone 4 mg (oral)	Dronabinol 2.5, 5.0, 10 mg; (oral)	Placebo hydromorphone (no placebo dronabinol condition)	Opioid dose held constant across all sessions	Limited evidence of dronabinol enhancement of hydromorphone on pain measures. Dronabinol 2.5 mg had a significant effect of thermal threshold and tolerance. Most pain measures did not show a significant difference between dronabinol+ hydromorphone and hydromorphone alone. No dose effect with dronabinol	GRADE rating “moderate”, indirect evidence as use of experimental pain. Higher doses of dronabinol (5 mg and 10 mg) also showed greater evidence of potential for abuse and adverse effects
Naef 2003	Experimental heat, cold, pressure, single and repeated transcutaneous electrical stimulation pain, randomized, placebo-controlled, double-blind, within-subject study	Healthy cannabis naïve volunteers (*n* = 12), 6 female, mean age 25 years	Four study sessions with at least seven days washout between sessions. Study medications co-administered, with pain measurements hourly for up to 8 h	Morphine 30 mg (oral)	Dronabinol 20 mg (oral)	Matched placebo capsule compared with THC alone, morphine alone or THC-morphine combination	No significant analgesic effect of dronabinol or morphine-dronabinol combination on heat, pressure and cold tests. Additive effect of morphine on transcutaneous electrical stimulation test	Potentiation of analgesia not observed in this experimental pain study, potential hyperalgesic effect of cannabinoids noted which may reduce analgesic effects of morphine	GRADE rating “moderate”, indirect evidence as use of experimental pain
Roberts 2006	Experimental thermal pain. Double-blind, four treatment within-subject design	Healthy volunteers (*n* = 13) with no recent opioid or cannabinoid use. Six female, aged 18–49 years	Four lab sessions; Dronabinol administered, 90 min later morphine administered, thermal pain measured 15 min after morphine administration	Morphine 0.02 mg/kg intravenous (1.4 mg dose for 70 mg adult) (i.e., sub- analgesic)	Dronabinol 5 mg (oral)	Placebo dronabinol capsule and placebo morphine injection (normal saline)	Not applicable (opioid dose held constant)	Combination of dronabinol and morphine did not have effect on pain intensity. The combination was reported to have a synergistic effect on affective response to pain (unpleasantness) compared with either drug alone (*p* = 0.012)	GRADE rating “moderate”, placebo-controlled blinded study, indirect evidence as use of experimental pain. Noted difficulties with extrapolation to clinical practice
*b. Controlled trials acute pain*									
Bebee 2021	Randomized, double-blind, placebo-controlled clinical trial (ACTRN12618000487213)	Adults with acute (<30 days duration) non-traumatic lower back pain (n=100). Median age 47 years, 44% female	48 h	Oxycodone (5 mg every 6 h, with additional rescue dosing as required)	CBD 400 mg (oral)	Color matched placebo prepared (medium chain triglyceride oil)	31/50 patients in the CBD group and 27/50 in the placebo group required oxycodone. Total oxycodone dose in the CBD group was 230 mg compared with 215 mg in the placebo group	Mean pain scores at 2 h were similar for the CBD (6.2 points; 95% CI, 5.5–6.9 points) and placebo groups (5.8 points; 95% CI: 5.1,6.6 points; absolute difference, –0.3 points; 95% CI, –1.3–0.6 points)	GRADE rating “high”
Levin 2017	Single-center randomized double-blind controlled trial (NCT02115529)	Patients scheduled for elective surgery under general anesthesia who had a preoperative risk of post-operative nausea or vomiting (*n* = 340). Mean age 49.8 years, 100% female	300 min or until discharge from post-anesthesia care unit	Specific opioid not reported, converted into OMEDD	Nabilone 0.5 mg (oral)	Matched placebo capsule	Morphine equivalents (mg) given in operating room: Nabilone 21.3 (SD 15.2) vs placebo 20.0 (SD 13.4), *p* = 0.40 Morphine equivalents (mg) post-surgery: Nabilone 5.8 (SD9.2) vs placebo 5.4 (SD 6.9) *p* = 0.62	No differences in pain score (out of a possible 10) between groups; Maximum pain score (at rest) Nabilone 3.17 (SD 3.15) vs placebo 3.68 (SD 3.25), *p* = 0.43 Maximum pain score (with movement) Nabilone 3.34 (SD 3.30) vs placebo 4.0 (SD 3.33), *p* = 0.92	GRADE rating “high”
Seeling 2006	Randomized double-blind controlled trial (two groups)	Prostate cancer patients <70 years, (all male) undergoing surgery (*n* = 105). 53 in intervention and 52 in control groups	From the day prior to surgery to 2 days post-operation	Pitrimide	Eight doses of Dronabinol 5 mg (oral) over 48 h (perioperatively)	Placebo dronabinol capsules	Median dose of pitrimide alone was 74 mg (IQR = 44–90 mg) compared with 54 mg (IQR = 46–88 mg) when administered with dronabinol	The median of the resting pain was not different between the groups (*p* > 0.1)	GRADE rating “high”, patients administered their own opioid doses
*c. Controlled trials cancer pain*									
Fallon 2017a^a^	Study 1: multisite (patients at 101 centers in 12 different countries) randomized, double-blind, placebo-controlled trial (NCT01361607)	Adults (*n* = 399) with advanced incurable cancer, unalleviated by an optimized opioid therapy. Mean age 59.8 years, 49% female	49 days (2 weeks after medication ceased)	Reported as OMEDD Patients were recruited on “stable opioid therapy”, not more than 500 mg OMEDD	1:1 nabiximols (Sativex®) oral mucosal spray (THC 27 mg/mL: CBD 25 mg/mL) or matching placebo. 14-day titration (3–10 spray/day to max tolerated dose), 35 days of medication provided in total	Placebo oral mucosal spray	No effect of nabiximols on; total OMEDD −9.35, 95% CI-18.81–0.012 (*p* =0 .053); maintenance OMEDD −3.63, 95% CI −10.80, 3.55 (*p* = 0.321); breakthrough OMEDD −4.17, 95% CI −8.76, 0.42 (*p* = 0.075). (note patients instructed to continue pain medication)	No differences in median percent improvement from baseline average pain NRS score: nabiximols 7.2% vs placebo 9.5% (median difference=−1.84%; confidence interval (CI): −6.19%, 1.50%; *p* = 0.274) Median treatment effect −0.02; 95% CI: −0.42, 0.38; *p* = 0.917)	GRADE rating “high” Significant effect of nabiximols on Subject Global Impression of Change, Patient Satisfaction Questionnaire and Physician Global Impression of Change
Fallon 2017b^a^	Study 2: 2-part enriched enrollment with randomized withdrawal design (“responders” randomized). Single-blind titration for 10 days followed by double-blind randomization to Sativex or placebo (NCT01424566)	Adults (*n* = 406 entered and 206 randomized) with advanced incurable cancer, unalleviated by an optimized opioid therapy, mean age 61.5 years, 43% female	49 days (2 weeks after medication ceased)	Reported as OMEDD. Patients were recruited on “table opioid therapy”, not more than 500 mg of OMEDD	Nabiximols (Sativex®) oral mucosal spray (THC 27 mg/mL: CBD 25 mg/mL). 10-day titration, “Responders” (15% pain score improvement) randomized. Treatment for 5 weeks	Placebo oral mucosal spray	No effect of nabiximols on: total OMEDD −7.1, 95% CI:23.9,9.7 (*p* = 0.405); maintenance OMEDD −8.9, 95% CI:19.7, 1.8 (*p* = 0.104); Breakthrough OMEDD 1.8, 95% CI:10.3, 14.0 (*p* = 0.769). (note patients instructed to continue pain medication)	Mean average pain scores increased from 3.2 to 3.7 in the nabiximols group and 3.1 to 3.6 in the placebo group, i.e., a worsening of equal severity in both treatment groups (estimated treatment effect −0.02; 95% CI: −0.42, 0.38; *p* = 0.917)	GRADE rating “moderate”, selection bias introduced through enrichment design
Johnson 2010^a^	Multicenter, randomized, double-blind, placebo-controlled, parallel-group trial. (NCT00674609)	Adults with cancer pain (*n* = 177), with inadequate analgesia despite chronic opioid dosing. Mean age 60.2 years, 56% female	2 weeks	Varied opioids reported as mg OMEDD (IQR)		Placebo oral mucosal spray (Baseline OMEDD for placebo group 120 (40–240))	Patients allowed to use breakthrough medication as needed; no change in median amount of breakthrough opioid medication in any group. Mean change in opioid dose from baseline Placebo −41.4 (SD 201.27), THC: 36.8 (SD 152.00); THC:CBD −3.5 (SD 108.44). Median change in all groups 0 mg	Change in pain score (out of 10) in favor of THC:CBD compared with placebo (−1.37 (*p* = 0.014)); the THC group change was not significant (−1.01 (*p* = 0.245)). The proportion of patients achieving a 30% reduction in pain was 43% (23/48) in the THC:CBD group, 23% in the THC group (12/45) and 21% in the placebo group (12/51)	GRADE rating “high”, placebo-controlled and randomized. No significant group differences were found in sleep quality or nausea scores or the pain control assessment
				120 (50–213)	THC 2.7 mg spray; mean of 8.47 sprays day				
				80 (30–180)	THC 2.7 mg:CBD 2.5 mg per spray; mean sprays 9.26/day				
Lichtman 2018^a^	Randomized, multisite double-blind, placebo-controlled study (12 countries) (NCT01262651)	Adults (*n* = 397) with advanced cancer-related chronic pain unalleviated by optimized opioid therapy. Mean age 59.9 years, 46% female	50 days if not entering extension study	Specific opioids not stated. Mean total daily opioid use at baseline 192.9 (SD 130.7) in the nabiximols group and 186.1 (SD 131.0) in the placebo group	Nabiximols (Sativex®) (THC 27 mg/mL: CBD 25 mg/mL), titration 14 days, maintenance 21 days. Max 10 sprays per day	Placebo oral mucosal spray	Nabiximols did not impact maintenance OMEDD (Estimated treatment difference [ETD] 1.46, 95% CI: −4.67, 7.60, *p* = 0.64), breakthrough OMEDD (ETD −1.84, 95% CI: −6.33, 2.66, *p* = 0.42) or total OMEDD (ETD −0.34, 95% CI: −8.26, 7.28, *p* = 0.93). Protocol stated that medications including opioids, should have been continued at stable doses if possible	Average pain score from baseline to end of treatment (primary endpoint): non-significant: 10.7% median improvement with nabiximols compared to 4.5% with placebo (*p* = 0.08). Nabiximols did not improve average pain NRS (*p* = 0.25) or worst pain NRS score (*p* = 0.68). Prespecified per-protocol analysis favored nabiximols over placebo (*p* = 0.04)	GRADE rating “moderate”, unclear blinding and randomization. Nabiximols was also associated with greater improvements than placebo in score on the Subject Global Impression of Change, Physician Global Impression of Change, and Patient Satisfaction Questionnaire
Lissoni 2014	Two groups (not randomized): cannabinoid tincture or melatonin (clinical trial registration not reported)	Adults (*n* = 26) with untreatable metastatic solid tumor (age and gender not stated)	Not stated	Oxycodone, median dose of 30 mg (10–60 mg), twice /day	Cannabis flos (19% THC) was given as infusion 100 ml (500 mg/L water) three times a day	Melatonin 20–100 mg	5/12 (42%) achieved control of pain without opioid dose increase compared to the control group where 2/14 (14%) achieved pain control	The number that achieved pain control was not significantly different between groups (5/12 in cannabis group and 2/5 in melatonin)	GRADE rating “low”, no-randomization, no allocation concealment described, greater disease progression in the cannabis group
Portenoy 2012^a^	Randomized, 4-arm double-blind, placebo-controlled, graded-dose study (NCT00530764)	Adults (*n* = 360) with advanced cancer and opioid-refractory pain, mean age 58 years, 48% female	5 weeks of medication administration	Median OMEDD 120	Nabiximols 1–4 Sprays	Placebo oral mucosal spray (Median OMEDD 120 mg)	No change in median amount of breakthrough opioid medication in any group. Note that patients were instructed to keep opioid dose constant so opioid-sparing effect on opioid dose could not be observed	OR 1.87 (*p* = 0.038) compared with placebo	GRADE rating “high”. Opioid composite measure showed better improvements in low dose group. Lower tolerability of THC:CBD in higher dose groups
				Median OMEDD 120	Nabiximols 6–10 Sprays			OR 1.70 (*p* = 0.079) compared with placebo	
				Median OMEDD 180	Nabiximols 11–16 Sprays			OR 1.16 (*p* = 0.622) compared with placebo	
Zylla 2021	Pilot randomized controlled trial comparing early cannabis (EC) to delayed start cannabis (DC)	Adults (*n* = 30) with stage IV cancer requiring opioids Patients in the EC group were similar to DC group with respect to mean age (57 (SD = 9) years vs 55 (SD = 13) years) and percentage female (47% vs 53%), respectively	3 months	Opioid type not specified. OMEDD measured using daily diary	Maintenance dose of 30–40 mg of THC and 30–40 mg of CBD per day, titrating up over 2–4 weeks	Early versus late start cannabis	EC group had stable opioid use; 3/9 in EC group and 4/9 in DC group increased OMEDD by ≥20%. Three patients in the EC group decreased their daily OMEDD by ≥20%	Mean pain scores remained similar between the two groups	GRADE rating “low” small sample with high attrition. Also examined dosing patterns: THC per patient each month was nearly twice that of CBD (average 34.3 mg THC vs 16.6 mg CBD)
*d. Controlled trials: chronic non-cancer pain*									
Abrams 2011	Clinical laboratory study of self-reported pain under observed conditions (also measured pharmacokinetic effects of concurrent administration) (NCT00308555)	Adults (*n* = 24) receiving chronic opioid treatment (mixed pain conditions). Mean age 45.1 years, 48% female	5 days	Morphine (mean dose 62 mg, *n* = 13) or oxycodone (mean dose 53 mg, *n* = 11)	Vaporized cannabis dose of 0.9 g of 3.56% delta-9-THC or as much as they could tolerate, administered three times a day	No comparator (single-arm study)	NA (opioid dose held constant)	Pain score: reduction from 34.8 (95% CI: 29.4, 40.1) on baseline to 24.1 (95% CI: 18.8, 29.4) on day 5 with morphine; and from 43.8 (95% CI: 38.6, 49.1) on baseline to 33.6 (95% CI: 28.5, 38.6) on day 5 with oxycodone. Significant reduction overall	GRADE rating “low”. No control arm, placebo effects cannot be excluded. No pharmacokinetic interaction observed. Cannabis inhalation produced a subjective “high”
Abrams 2020	Randomized double-blind, two group crossover design (NCT01771731)	Adults with sickle cell disease with chronic pain (*n* = 23), 21 of whom were using opioids. Mean age, 37.6 years; 56% female	5 inpatient days with 30-day washout followed by another 5 inpatient days	Hydromorphone, oxycodone, hydrocodone, morphine sulfate, fentanyl, methadone and oxymorphone	Cannabis plant material containing 4.4% THC and 4.9% CBD which were vaporized and inhaled 3 times per day	Vaporized placebo cannabis	The mean (SD) difference in log OMEDD dose between the cannabis and placebo periods in this value was not significant (2.05 [0.21] vs 2.09 [0.22]; *p* = 0.20)	The mean (SD) difference in pain rating assessment using the VAS data between the active and placebo groups were not significant on any day	GRADE rating “low”; small sample size and unclear blinding procedures and crossover design
De Vries 2016	Randomized, single-dose, double-blind, placebo-controlled, two-way crossover study (NCT01318369)	Adults aged 18 and above with chronic abdominal pain from chronic pancreatitis (*n* = 24, 12 of whom were taking opioids). Mean age of sample 52 years, 9 of 24 patients were female	6 h	Pethidine; tramadol and codeine (patients’ usual analgesic medication)	Dronabinol 8 mg	Diazepam 10 mg	The pharmacokinetic parameters of THC were similar between opioid and non-opioid users. Opioid dose requirements were not an outcome of the single-dose study	Primary analysis showed no treatment effect of THC. When only patients on opioids were considered, the mean VAS pain score at 2 h was similar for patients on in THC arm (2.917, SD 2.205) and the diazepam (active placebo) arm (2.53, SD 1.702)	GRADE rating “moderate” downgraded due to small sample size and crossover design. Additional data provided by authors
De Vries 2017	Randomized, single-dose, double-blind, placebo-controlled, two-way crossover study (NCT01562483 and NCT01551511)	Two clinical trials where the samples were combined: (1) Adults with painful chronic pancreatitis (CP) *n* = 23, and (2) adults with chronic postsurgical abdominal pain (PSP), *n* = 27, mean age 52.9 years, 50% female	61 days	Codeine, tramadol, oxycontin, fentanyl, and morphine (patients usual medicines)	Dronabinol tablet, increased to 8 mg three times a day over 10 days, with the option to reduce to 5 mg if 8 mg is not tolerated. Those not tolerating 5 mg three times a day were withdrawn	Matched dronabinol placebo tablet	Not reported. Patients were asked to continue taking their medications (including analgesics) according to prescription	Primary analysis (all patients) VAS mean scores did not differ between THC and placebo. For patients on opioids: (1) CP group (*n* = 20) mean VAS on day 49 was 2.05, SD 2.65) with THC compared with 2.94, SD 2.10 for placebo (*p* = 0.4). (2) PSP (*n* = 17), mean VAS on day 49 for placebo was higher (5.51, SD 2.27) compared with THC (2.9, SD 2.35), *p* = 0.03	GRADE rating “moderate”, small sample size and high attrition in the active arm for the CP group. Additional data provided by authors
Narang 2008	Phase 1: randomized, single-dose, double-blind, placebo-controlled, crossover trial. Primary outcome measure Total Pain Relief score (Phase 2 extension study in Table 2b) (NCT00153192)	Adults taking opioids for chronic pain; BPI ≥ 4 (*n* = 30). Pain diagnosis: neuropathic (*N* = 7), nociceptive (*N* = 7), mixed neuropathic and nociceptive (*N* = 11), and uncategorized (*N* = 5). Mean age 43.3 years, 53% female	Phase 1: Three, 8-h lab sessions with three days washout.	OMEDD mean 68.1 mg (SD: 57.2, range 7.5–228) (mix of oxycodone, morphine, methadone hydrocodone, hydromorphone)	Dronabinol 10 and 20 mg	Matched placebo dronabinol capsule	One subject took rescue pain medication in all 3 conditions (placebo, dronabinol 10 mg, and dronabinol 20 mg), one subject on both placebo and 10 mg condition, 6 subjects during their placebo condition only	In single-dose studies 10 and 20 mg dronabinol significantly increased the amount of analgesic relief reported. Total pain relief: 31.1 in placebo: 39.7 with dronabinol 10 mg: 41.7 with dronabinol 20 mg	GRADE rating “moderate”, randomized and placebo-controlled, small sample
*e. Observational studies*									
Aviram 2020	Prospective observational cohort study	Adults with any form of chronic non-cancer–related pain (*n* = 829, 66% retained at 12 months). Average age 47 years, 43% female	12 months following treatment initiation	Not specified, mixture of weak (*n* = 118) and strong (*n* = 56) opioids at baseline	Varied products (74% THC dominant). Overall reported monthly MC dose increased from 20 ± 20–20 g at T1 to 30 ± 20–30 at T12 (*χ*^2^ (4) = 1250.32, *p* < 0.001).	NA	42% reduction [27 mg OMEDD (95% CI: −34.89, 18.56) reduction (*p* < 0.001)). Opioid cessation at 12-month follow-up: 24% using weak and 20% using strong opioids; *χ*^2^ (5) = 27.3, *p* < 0.001; *χ*^2^ (5) = 21.9; *p* < 0.001, respectively	Not reported by opioid status	GRADE rating “low” large observational cohort. In the same cohort, of those not taking opioids at baseline 9% started weak opioids and 10% started strong opioids
Bellnier 2018	Observational pre-post study	Adults (*n* = 29) with chronic pain (*n* = 3 cancer-related, *n* = 26 non-cancer pain, 20 with spinal cord injury with spasticity). Average age was 61 years (±10 years), and 65% of the sample were female	3 months	Not specified, calculated at OMEDD	10 mg capsules of 1:1 THC and CBD taken orally every 8–12 h. Vapor pen inhaler of THC/CBD (20:1, 2 mg THC per 0.1 mg CBD) 1 to 5 puffs every 15 min until relief was achieved and use every 4–6 h as needed	Pre-post-	Opioid consumption was reduced from 79.94 (range 0–450) to 19.65 (range 0–150) morphine equivalents per day (*p* < 0.05). Most (*n* = 26) completely discontinued opioid use, and the remaining patients reduced their doses by approximately 75%	Paroxysmal pain decreased from 6.76 to 2.04 (*p* < 0.0001); surface from 4.20 to 1.30 (*p* < 0.0001); deep from 5.87 to 2.03 (*p* < 0.0001); and unpleasant rating declined from “miserable” to “annoying” after 3 months therapy	GRADE rating “low” observational data Quality of life EQ-5D scores (range 0 to 100) improved from 36.08 ± 19.85 at baseline to 64.43 + 19.15 after 3 months treatment (*p* < 0.0001)
Capano 2020	Prospective, observational cohort study	Adults (*n* = 113) with mod-severe chronic pain for at least 3 years, and stable opioids for at least 1 year (*n* = 113, 74% retained at 8 weeks, aged 30–65 years (mean 56.1 years), 68% female	4 and 8 weeks	Not stated, requirement to be taking at least 50 mg OMEDD for 12 months prior to enrollment	15.7 mg CBD, 0.5 mg THC, 0.3 mg cannabidivarin (CBDV), 0.9 mg cannabidiolic acid (CBDA), 0.8 mg cannabichrome (CBC), and >1% botanical terpene blend	NA	OMEDD not captured. Fifty of the 94 (53.2%) participants using the CBD hemp extract were able to reduce opioid medications at week 8. Of the fifty who reduced, two ceased completely	Baseline pain (PEG) scale at (6.5 [95% CI: 6.16–6.81], 4 weeks 5.9 [95% CI: 5.55–6.25] and 8 weeks,5.7 [95% CI: 5.31–6.12], p =0.006) (12% reduction in pain, [30% reduction considered clinically significant])	GRADE rating “low” observational data with no control
Habib 2018	Retrospective cohort study	Adults aged 18 and above with fibromyalgia (*n* = 26), 19 female patients (73%), mean 37.8 ± 7.6 years	Median cannabis duration 3 months	Codeine, tramadol, oxycodone, fentanyl or buprenorphine. Varied doses	The mean dose of medical cannabis was 26 ± 8.3 g per month	NA	4/4 patients on weak opioids at baseline, ceased while taking cannabinoids (*p* = 0.055); 15/20 patients on strong opioids ceased while taking medical cannabinoids (*p* = 0.000)	Not reported by opioid status	GRADE rating “very low”, small retrospective cohort with no control group, short follow-up
Haroutounian 2016	Prospective, observational cohort study	Adults (*n* = 73/274) 18 years + above 18 with chronic pain, 73 prescribed opioids. Mean age 51.2 years, 62% female	6 months	Morphine, oxycodone, fentanyl, hydromorphone, buprenorphine, methadone and tramadol.	Smoked (THC 6–14%, CBD 0.2–3.8%) and oral (THC 11–19%, CBD 0.5–5.5%). The mean (SD) monthly prescribed amount of cannabis was 43.2 (17.9) g (any formulation)	NA	32/73(44%) ceased opioids, (*p* < 0.001). Median OMEDD among participants receiving opioids at follow-up (*n* = 41) decreased from 60 mg (95% CI: 45–90) to 45 mg (95% CI: 30-,90); (*p* = 0.19, Mann–Whitney)	Pain outcomes not available for the subsample on opioids	GRADE rating “low”, non-randomized single-arm open-label study
Hickernell 2018	Retrospective cohort study	Adults (*n* = 243) who had total knee or hip arthroplasty. Mean age 62.3 years, 64% female	Mean length of stay 2–3 days	Oral oxycodone 10 mg up to three doses mg plus immediate release oxycodone 5–10 mg mg as required	Dronabinol 5 mg twice a day during hospital stay (*n* = 81)	Patients who did not receive dronabinol (*n* = 162) over the same time period	No significant difference in OMEDD or total OME dose/length of stay. Significantly lower total OME consumption during stay: Dronabinol group 252.5 mg ± 131.5 mg, control group 313.3 mg ± 185.4 mg. *p* = 0088	No significant difference on pain scores between the groups on any day post-surgery	GRADE rating “low” non-randomized retrospective study. Mean length of stay lower for the dronabinol group compared with control (2.3 ± 0.9 vs 3.0 ± 1.2 days, *p* = 0.02)
Hoggart 2015	Open-label extension study from 2 clinical trials across 66 study sites (38 centers in six countries)	Adults (*n* = 380) with peripheral neuropathic pain, mean age 57.8 years, 47% female	38 weeks	Strong and weak opioids	THC/CBD oral mucosal spray (2.7 mg of THC and 2.5 mg of CBD per spray) max 24 sprays per day	NA	No change in the proportion of the whole sample taking strong opioids (56/380 at baseline and 57/380 at follow-up) or other opioids (118/380 at baseline to 123/380 at follow-up) following cannabinoid use	Data on other outcomes not provided by opioid use status	GRADE rating “moderate”, non-randomized sample. Rigorous data collection
Lynch 2002	Observational case series	Adults with pain conditions (*n* = 3) (peripheral neuropathy, multiple sclerosis, lower back pain), Aged 35–47 years, 33% female	1–9 month observation period	Morphine (varied doses)	Smoked cannabis plant, unknown content	NA	Mean baseline morphine dose 195 mg (SD 147 mg) compared with mean 35 mg (SD 31 mg) after commencing smoked cannabis. Opioid dose reduction or cessation in each case	Improved pain control described with patients either reducing or ceasing morphine dose	GRADE rating “very low“, unblinded observational study
Maida 2008	Prospective observational study	Adults with advanced cancer (*n* = 112), 47 of whom were treated with nabilone (mean age 67 years, 38% female)	30 days	Nabilone group baseline OMEDD 60.3 mg (SD 64.6); comparison group OMEDD 67.3 mg (SD 101.0)	Nabilone, mean of 1.79 mg daily	People with advanced cancer who were not treated with nabilone	Log OMEDD in nabilone group 3.8 mg compared with 4.3 mg in the untreated group (*p* = 0.016), remained significant after adjusting for baseline symptom level and propensity score	Pain score in nabilone group 3.7 compared with 5.0 in the untreated group (*p* = 0.003); remained significant after adjusting for baseline symptom level and propensity score with pain score of 3.0 in the nabilone group and 5.5 in the comparison group (*p* < 0.001)	GRADE rating ”low”. Nabilone prescribing based on symptom-related distress on the initial consultation, leading to selection bias, but managed with propensity scoring
Maida 2017	Observational case series	Adults with pyoderma gangrenosum (*n* = 2) on opioids. Female (50 years) and male (76 years)	6–25 days	Opioid analgesic type not specified	Topical cannabinoid oil THC:CBD 5:6 mg/mL or THC:CBD 7:9 mg/mL	NA	Mean Baseline OMEDD 26.7 mg (SD 0.9), Mean follow-up 6.4 mg (SD 8.7)	Mean pain at baseline 8.6, mean pain at follow-up was 2.6 (70% reduction, i.e., clinically meaningful reduction)	GRADE rating “very low”, very small case series
Maida 2020	Observational case series	Two adults (aged 86 and 69, both female), with painful and non-healing leg ulcers, of greater than 6 months duration	57–68 days	Case 1: Codeine (with acetaminophen), Case 2 188 mg oral morphine equivalents (opioid type not stated)	Topical cannabinoid product THC < 1 mg/mL, CBD 3.75 mg/mL	NA	Both patients ceased opioids	Not reported, opioid requirements used as proxy for pain	GRADE rating “very low”, very small case series
Narang 2008	Open-label extension following randomized, single-dose, double-blind, crossover trial (Table 2d)	Patients on opioids for chronic pain; BPI ≥ 4 (*n* = 28). (see Table 2d for participant characteristics)	Four weeks	OMEDD mean 68.1 mg (SD 57.2, range 7.5–228) (mix of oxycodone, morphine, methadone hydrocodone, hydromorphone)	Flexible dose schedule; dronabinol 5 mg daily − 20 mg three times a day.	NA	Opioid dose not reported	Mean baseline NRS of 6.9 compared with mean NRS of 5.2 after 4 weeks of dronabinol (24% reduction in pain). Statistically significant reduction, but does not meet the 30% reduction in pain to be clinically significant	GRADE rating “low”. Improvements (*p* < 0.05) in sleep, energy, pain relief, and social functioning. Lack of placebo control means effects may be non-specific/placebo
Rod 2019	Open-label prospective opioid taper study	Patients with chronic pain (*n* = 600), on opioid doses of 90–240 mg (age and gender not reported)	Six months	Mean OMEDD 120 mg (Range 90–240 mg)	CBD and THC (4–6%). Doses related directly to the opioid taper: 0.5 g/day for each 10% reduction in opioid dose, as needed by sublingual, oral or inhalation by vaporization	NA	156 patients (26%) ceased opioids, and a further 329patients (55%) reduced opioid use by an average of 30%. Cannabis use among these patients ranged from 1–3 g/day	Pain not quantified. One patient increased opioid intake; all other patients expressed satisfaction with their pain control, sleep and quality of life	GRADE rating “low”, evidence-based online psychological support provided (e.g., cognitive behavioral therapy and mindfulness)
Safakish 2020	Prospective observational cohort study	82/751 chronic pain patients, who were using opioids. Mean age of 49.6 years, 57% female	12 months	Mixed opioids, converted to oral morphine equivalent doses	7% to 29% THC and/or CBD.	NA	Baseline (*n* = 82) OMEDD 26.2 (SD 48.1), Month 1 (*N* = 67) 12.1 (SD 44.7), month 3 (*N* = 26) 3.3 (SD 8.6), month 6 (*n* = 9) 3.0 (SD 6.5) month 12 (*n* = 4) 1.4 (SD 0.1). *p* < 0.001	Not reported by opioid status	GRADE rating “very low”, open-label single harm study with high attrition
Schneider-Smith 2020	Retrospective matched cohort study	Adults with traumatic injury: 33 cases (mean age 39.9 years, 76% male) and 33 matched controls (mean age 30.0 years, 30% female)	48–96 h after admission	Not stated, opioid use reported in OMEDD	Dronabinol (usually 5–10 mg twice a day)	Usual care without dronabinol	OMEDD reduction in group dronabinol (−79 mg (SD20), *p* < 0.001), OMEDD for controls unchanged from baseline (−9 (18) mg, *p* = 0.63)	Adjunctive dronabinol reduce pain scores. Average change in pain scores (NRS) were similar between cases and controls (−0.4 vs −0.6, *p* = 0.78)	GRADE rating “low”, non-randomized retrospective study
Takakuwa 2020	Retrospective cohort study	Adults with low back pain (*n* = 61) who were prescribed opioids. Mean age 50.1 years, (range = 49–86), 38% female	Data extracted from 1997–2019 from a database of more than 3000 patients	Median OMEDD 21 mg/day (range = 1.1–500).	Variable products reported in grams per day. Median cannabis use 1.45 g/day (range = 0.01–18.7 g). Most smoked (44/61 (72%)).	NA	31/61 ceased, 9 reduced and 15 increased their opioids. Median OMMED reduction 9.0 mg (IQR = 24–6), sign test: *p* = 0.004, Wilcoxon sign rank test: *p* = 0.09). Subgroup analysis: chronic use (media*n* = 21, IQR = 90–5) intermittent opioid use (media*n* = 5.8, IQR = 12–6)	Not reported	GRADE rating “very low”, small retrospective cohort study
Yassin 2019	Prospective observational crossover study	Adult (*n* = 31) with low back pain and fibromyalgia already treated with oxycodone and duloxetine. Mean age 33.5 years., 90% female	6 months	Oxycodone 5 mg/Naloxone 2.5 mg twice a day (minimum 12 months of standard analgesic therapy prior to trialing medical cannabinoids)	Medical cannabinoids (smoked or vaporized) 1:4 THC to CBD. The THC levels were less than 5%. The dose of cannabinoid was 20 grams per month for 3 months, with the option to increase to 30 g/month thereafter	Baseline status on standard analgesic therapy	Not reported	Mean VAS score at baseline 8.1(SD 1.4); 3 months: 5.3 (SD 1.3); 6 month: 3.3 (SD 2.2) *p* < 0.0001. 60% reduction in pain at 3 months, i.e., clinically meaningful reduction	GRADE rating “very low”, small sample, open-label single-arm study; selection bias as only those that did not respond to standard therapy included

*GRADE* Grading of Recommendations Assessment, Development and Evaluation, *CBD* cannabidiol, *OME* oral morphine equivalent, *OMEDD* oral morphine equivalent daily dose, *IQR* interquartile range, *SD* standard deviation, *THC* delta-9-tetrahydrocannabinol, *NRS* numerical rating scale, *BPI* Brief Pain Inventory, *VAS* visual analog scale.

^a^Data extracted from publication in addition to clinialtrials.gov.

#### Clinical trials—experimental pain

Five laboratory-based studies in healthy volunteers (*n* = 82) examined pain responses with co-administered opioids and cannabinoids using double-blind within-patient study designs (Table 2a). Four studies examined oral dronabinol (2.5–20 mg) [59–62] and one examined smoked cannabis [63]. Inconsistent outcomes were observed; two studies found evidence of increased pain, two found some measures of decreased pain, and one study found effects of cannabinoids on pain “unpleasantness” but not pain ratings. One study found low dose dronabinol (2.5 mg) decreased the analgesic effects of oxycodone as measured with a pressure algometer with no effect of 5 or 10 mg dronabinol on analgesic outcomes [61]. Another study noted potentially hyperalgesic effects of cannabinoids [59]. This was in contrast to the analgesic effect observed on pain threshold and tolerance with a cold pressor test when smoked cannabis was administered with 5 mg oxycodone compared oxycodone or cannabis alone, although effects were not found on measure of outcomes of pain intensity or bothersomeness [63]. Dunn et al. [62] demonstrated analgesic effects from dronabinol 2.5 mg when co-administered with hydromorphone on thermal pain measures, but not with higher doses of dronabinol, or on other measures of pain. Roberts et al. [60] found that the co-administration of dronabinol and morphine resulted in reduced pain “unpleasantness” compared to either drug alone. Three experimental studies included measures of abuse liability, and found that smoked cannabis and dronabinol may increase the abuse liability ratings of oxycodone and hydromorphone using measures such as ratings of feeling high and drug liking [61–63].

#### Clinical trials—acute pain

Three double-blind randomized controlled trials (*n* = 545) examined the opioid-sparing effects of CBD in acute pain [64–66]. Nabilone and dronabinol were examined in acute post-operative pain and CBD in acute low back pain (<30 days duration). No benefit on opioid dose requirements or analgesic outcomes was identified (Table 2b).

#### Clinical trials—cancer pain

Seven controlled trials (1795 participants) investigated the opioid-sparing effect of cannabinoids in patients with different forms of cancer pain. One small, non-randomized study found a non-significant effect of cannabis on pain control [67], and a second pilot found no effect of medical cannabis on pain, but an increase in opioid dose in a group that received delayed cannabis [68] (Table 2c). The remaining studies were all larger single or double-blind randomized trials. Five randomized controlled trials (reported in four publications) examined THC and nabiximols compared to placebo in patients with cancer pain who were taking opioids [69–72]. Two studies found improved analgesia with nabiximols compared to the placebo. Johnson et al. [69] found no effect of nabiximols on breakthrough opioid dose requirements. Portenoy et al. [70] conducted a dose-ranging study, and a significant analgesic effect was only found in the lowest dose group, with poorer tolerability observed for higher doses. The remaining three studies found no benefit of adding cannabinoids on their primary outcome of analgesia. Although Lichtman et al. [72] did not find a significant effect of cannabinoids on pain in an intention to treat analysis, the per-protocol analysis did find a significant effect (Table 2c). Four of seven studies required maintenance opioid doses to be kept stable [70–72]; five studies measured breakthrough opioid doses requirements as an outcome with no evidence of a difference found [69–72]. No cancer pain studies included measures of abuse liability.

**Fig. 2:**
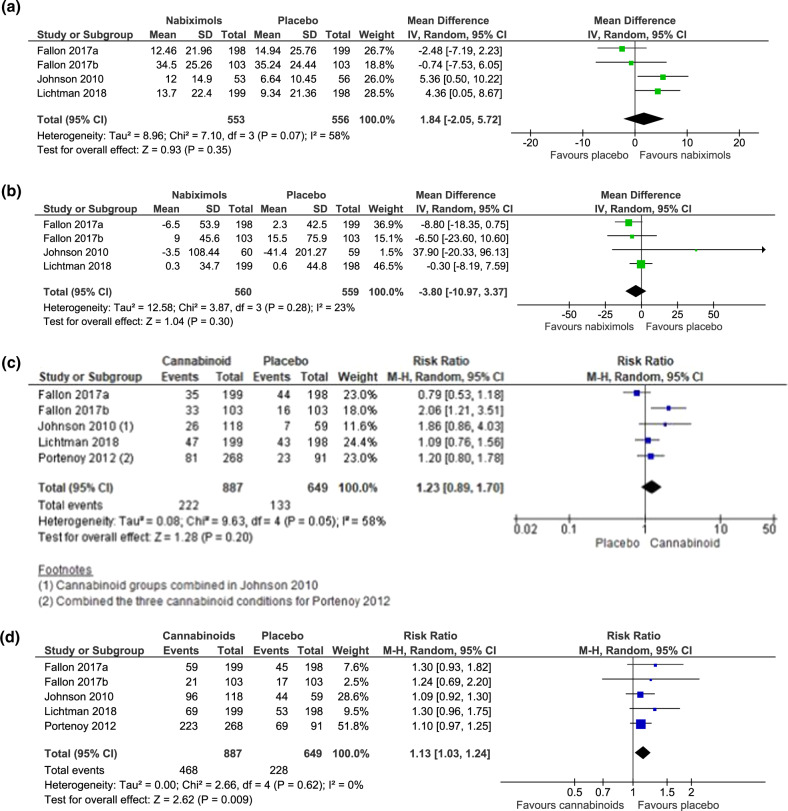
Opioid-sparing outcomes from clinical trials in people with cancer pain. Meta-analysis comparing cannabinoids with placebo on outcomes of **a** percent improvement in pain score, **b** change in mean total Oral Morphine Equivalent Daily Dose (OMEDD), **c** serious adverse events from baseline, and **d** adverse events excluding serious adverse events, in clinical trials of people with cancer pain.

Meta-analyses were possible on the outcomes of change in mean total oral morphine equivalent daily dose (OMEDD) from baseline (*n* = 4 studies), percent change in pain score from baseline (*n* = 4 studies) and adverse events (*n* = 5 studies). Meta-analysis of four studies (*n* = 1119 participants) found no effect of nabiximols on change in OMEDD (Mean difference −3.8 mg, 95% CI −10.97, 3.37, *I*^2^ = 23%) (Fig. 2a). Four studies (1109 participants) found no effect of nabiximols on percentage change in pain scores (mean difference 1.84, 95% CI −2.05, 5.72, *I*^2^ = 58%) (Fig. 2b). Five studies (1536 participants) examined serious adverse events and found no difference in events with cannabinoids compared with placebo (risk ratio [RR] 1.23, 95% CI 0.89, 1.70, *I*^2^ = 58%) (Fig. 2c). Five studies (1,536 participants) examined adverse events other than serious adverse events and found more non-serious adverse events with cannabinoids compared with placebo (RR 1.13, 95% CI 1.03, 1.24, *I*^2^ = 0%) (Fig. 2d).

#### Clinical trials—chronic non-cancer pain

Five clinical trials (139 participants, Table 2d) examined the effects of dronabinol [73–75] and smoked cannabis [76, 77] in patients with chronic non-cancer pain. Most studies had short observation periods (5 h to 5 days) [74–77], and used crossover designs [73–76]. Opioid dose was an outcome in one study, with no difference between smoked cannabis and placebo [76]. All five studies reported on analgesic outcomes with conflicting findings. A single-arm open-label study (with no comparison group) recruited people with mixed types of chronic non-cancer pain (*n* = 24) who were prescribed opioids, and found significant overall reductions from baseline pain ratings following co-administration of cannabinoids [77]. In contrast, a double-blind crossover study in sickle cell patients found no significant differences analgesia effects between placebo and vaporized cannabis [76]. Two studies recruited patients with chronic pancreatitis and found no effect of dronabinol on pain measures compared with placebo [73, 74]. A sub-analysis in patients with chronic postsurgical abdominal pain found lower pain among those who received dronabinol compared with placebo [73]. A single-dose study in patients with mixed-chronic pain conditions, found dronabinol 10 and 20 mg was associated increased analgesia compared with placebo [75]. These studies did not include measures of abuse liability.

#### Clinical studies—observational

Seventeen observational studies (*n* = 2674) examined the opioid-sparing effects of cannabinoids; three small retrospective case series of two to three patients each [78–80], two retrospective cohort studies [81, 82], two retrospective matched cohort studies [83, 84], and ten prospective observational cohort studies [85–93], including two open-label extension studies [75, 93] (see Table 2e). Two retrospective matched cohort studies examined acute analgesia with traumatic injury [83] and joint arthroplasty [84]. Both found no difference in pain scores, but reduced opioid consumption on at least one measure. For pain management following joint arthroplasty, there was no change in daily opioid dose with dronabinol administration, but a reduced total opioid consumption due to significantly shorter hospital stays in the dronabinol group [84]. One study compared those prescribed nabilone with those that had not received it, using propensity scoring to adjust for the greater severity of the nabilone prescribed group [89]. The remaining observational studies did not have control conditions and examined opioid use in patients with a range of different types of chronic non-cancer pain. Seven studies reported on the outcome of OMEDD after commencing medical cannabinoids, with reductions from 9 to 140 mg OMEDD reported (Table 2b). Four studies quantified the reduction in pain scores, which ranged from 12% to 70%, with two studies exceeding the minimum threshold of a 30% reduction in pain to be clinically meaningful. Meta-analysis was possible for studies that reported the proportion of patients who reported opioid reduction or cessation; eight studies reported the proportion of patients who ceased opioids (range 2–100%), with a pooled prevalence of 0.39 (95% CI 0.15, 0.64, *I*^2^ = 95.47%) (Appendix 5a). Seven studies reported on the proportion of patients reducing opioid use (range 44–100%) with a pooled prevalence of 0.85 (95% CI 0.64, 0.99, *I*^2^ = 92.82%) (Appendix 5b). Statistically significant heterogeneity was identified in both meta-analyses.

### Quality ratings of clinical studies

The clinical studies were rated using the GRADE criteria. Of the clinical trials, five laboratory studies provided moderate evidence, three clinical trials in acute pain provided high quality evidence, six clinical studies provided low-high quality evidence in cancer pain, and five studies in chronic non-cancer pain were assessed as low-moderate quality. The seventeen observational studies were assessed to be low to very-low-quality evidence (Table 2).

#### Ongoing clinical trials

We identified 15 registered clinical trials which, based on published protocols and clinical trial registry entries, may provide important data for future updated reviews (Appendix 6).

## Discussion

The current update represents the largest synthesis of studies examining the opioid-sparing effects of cannabinoids, with double the number of preclinical studies, four times as many clinical studies and more than six times the number of participants (>5000) compared to our earlier review [3], reflecting the rapid growth of clinical research in this area.

Most preclinical studies found synergistic effects with opioids and cannabinoids co-administration, predominantly with mixed CB1/CB2 agonists such as delta-9-THC, though effects varied with different cannabinoids, opioids and pain assays. Meta-analyses (with one addition preclinical study since 2015) demonstrated that morphine dose required to produce an equivalent analgesic effect was 3.5 times lower when co-administered with delta-9-THC, consistent with the previous review [3]. This effect would be clinically meaningful if replicated in well-controlled clinical studies. However, preclinical studies often have larger effect sizes, attributed to the reduced heterogeneity compared to clinical populations [94]. This body of preclinical research may help to identify specific cannabinoids and mechanisms that underlie an opioid-sparing effect, with the most consistent effects observed with mixed CB1/CB2 agonists, and evidence of potential antagonistic effects between CB1 agonist and mu receptor agonists in models of mechanical hyperalgesia.

A rapidly growing number of clinical studies have measured opioid-sparing endpoints, though findings were inconsistent. The highest quality studies were conducted in patients with cancer pain, where meta-analysis of four studies did not find significant effects on opioid dose or analgesia. Conflicting findings were found in studies of experimental pain, and in patients with chronic non-cancer pain. Further studies are needed to clarify the results found here given the small number of studies.

A limited number of controlled studies demonstrated benefits of combining cannabinoids with opioids for analgesia. Experimental pain studies found cannabinoids improved [62, 63] and worsened [61] analgesia. These effects were not dose dependent, with significant effects seen with lower but not higher doses of delta-9-THC. Opioid-sparing effects were not seen in well-conducted RCTs with acute pain, or in meta-analyses of RCTs in cancer pain, and studies that did find positive effects have important limitations such as no control group [77], small sample sizes [67, 75], and the mixed quality of the study design. Furthermore, some RCTs instructed patients to continue their pain medication in the same doses, which may preclude identifying a change in opioid dose [70–73, 77], although changes in breakthrough opioid requirements were a secondary outcome in six studies [69–72, 75]. Some clinical studies demonstrated beneficial effects of opioid and cannabinoid co-administration on other outcomes such as sleep, and functioning in chronic pain patients [75, 77]. Conflicting results were found between preclinical studies and clinical trials on measure of abuse liability. Evidence of reduced abuse liability was found in some animal models, which contrasted directly with evidence of increased drug liking and subjective effects in human studies.

Finally, observational studies had methodological concerns including small sample sizes (several observational studies included in meta-analysis had two to three patients), no control groups or blinding, selection bias, and were likely to have been impacted by expectancy effects.

Although our review is much broader, we have drawn similar conclusions to earlier reviews. For example, a review of cross-sectional surveys and cohort studies, representing lower quality evidence, found large reductions in opioid doses, though study designs prevented the drawing of causal conclusions [95]. A later review with five randomized trials with patients with chronic pain and 12 observational studies further concluded that there was uncertainty in the evidence [96], although this review considered a substantially smaller number of clinical trials than we consider. Future studies may benefit from focusing on populations with higher opioid tolerance, or higher motivation to reduce opioid doses, where clinical benefits may be greatest [97]. Standardization of outcomes for opioid-sparing research may assist with harmonization of outcome measures and support meta-analysis with future clinical trials [2].

Despite the inclusion of a larger number of studies, and the increased size and quality of clinical trials in recent years, our conclusions have not changed substantially from our earlier review. Nevertheless, we did identify 15 registered clinical trials indicating that this continues to be an active area of research in which the science is likely to continue to evolve.

In conclusion, preclinical studies support the opioid-sparing effect of delta-9-THC and other mixed CB1/CB2 agonists. Observational studies support the opioid-sparing potential of cannabinoids. However, findings from clinical trials provide conflicting results that may highlight important areas for future research. These include identifying optimal doses and populations who may experience benefits with cannabinoids. With numerous clinical trials currently underway, we will update our review, as higher-quality data may enable stronger conclusions to be made.

## Supplementary information

Online appendices
