# Supplementary material for: Opioid-sparing effect of cannabinoids for analgesia: an updated systematic review and meta-analysis of preclinical and clinical studies
Source: Neuropsychopharmacology. 2022 Apr 22;47(7):1315–30. doi: 10.1038/s41386-022-01322-4 (PMC9117273; doi:10.1038/s41386-022-01322-4)
Supplement: Supplementary file 1 — Online appendices [file 41386_2022_1322_MOESM1_ESM.pdf]

Online Appendices: Opioid-sparing Effect of Cannabinoids: A Systematic Review and Meta-analysis

Suzanne Nielsen, Louisa Picco, Bridin Murnion, Bryony Winters, Justin Matheson, Myfanwy Graham, Gabrielle Campbell, Laila Parvaresh, Kok-Eng Khor, Brigid Betz-Stablein, Michael Farrell, Nicholas Lintzeris, Bernard Le Foll

Corresponding author : Suzanne.nielsen@monash.edu

Please contact to request any materials used in the review. Data extracted from included studies are contained in Tables 2, Figure 1 & 2 and Appendix 4.

Appendix 1: Embase search terms 2
Appendix 2. Excluded Studies with reasons for exclusion 3
Appendix 3. PRISMA diagram showing study identification 7
Appendix 4. Preclinical studies 8
Appendix 5. Proportion of participants in observational studies reporting cessation and reduction in opioid use 16
Appendix 6: Registered clinical trials, where results are not yet available 17

Appendix 1: Embase search terms

1.	exp opiate/
2.	analgesic agent/
3.	(opioid* or opiate* or opium).tw.
4.	1 or 2 or 3
5.	cannabinoid/
6.	cannabinoid*.tw.
7.	Cannabis/
8.	Cannabis.tw.
9.	sativex.tw.
10.	nabiximols/
11.	nabiximol*.tw.
12.	cannabinol/
13.	cannabinol.tw.
14.	tetrahydrocannabinol/
15.	tetrahydrocannabinol.tw.
16.	5 or 6 or 7 or 8 or 9 or 10 or 11 or 12 or 13 or 14 or 15
17.	exp Pain/
18.	pain.tw.
19.	opioid sparing.tw.
20.	opioid dose.tw.
21.	antinociceptive agent/
22.	antinociceptive.tw.
23.	17 or 18 or 19 or 20 or 21 or 22
24.	4 and 16 and 23
25.	limit 24 to yr="2015 -Current"

Appendix 2. Excluded Studies with reasons for exclusion

**Author, year**	**Title**	**Reason for exclusion**
Abraham, A. D.; Leung, E. J. Y.; Wong, B. A.; Rivera, Z. M. G.; Kruse, L. C.; Clark, J. J.; Land, B. B. (2020)	Orally consumed cannabinoids provide long-lasting relief of allodynia in a mouse model of chronic neuropathic pain	Wrong intervention
Aleissa, M. M.; Ahern, K. L.; Stern, G. M. (2020)	Peri-operative opioid and sedation requirements in patients who use marijuana and are undergoing total knee or total hip arthroplasty: A retrospective study	Wrong study design
Arboleda, M. F.; Dam, V.; Prosk, E.; Dworkind, M.; Vigano, A. (2018)	Cannabis-Based Medications: The Future Co-analgesics of Choice for Cancer Patients?	Unable to confirm details with author
Balash, Y.; Bar-Lev Schleider, L.; Korczyn, A. D.; Shabtai, H.; Knaani, J.; Rosenberg, A.; Baruch, Y.; Djaldetti, R.; Giladi, N.; Gurevich, T. (2017)	Medical Cannabis in Parkinson Disease: Real-Life Patients' Experience	Wrong study design
Barlowe, T. S.; Koliani-Pace, J. L.; Smith, K. D.; Gordon, S. R.; Gardner, T. B. (2019)	Effects of Medical Cannabis on Use of Opioids and Hospital Visits by Patients With Painful Chronic Pancreatitis	No cannabis dose reported
Bauer, F. L.; Donahoo, W. T.; Hollis, H. W.; Tsai, A. G.; Pottorf, B. J.; Johnson, J. M.; Silveira, L. J.; Husain, F. A. (2018)	Marijuana's Influence on Pain Scores, Initial Weight Loss, and Other Bariatric Surgical Outcomes	Wrong study design
Bekker, A. (2018)	Cannabis use and non-cancer chronic pain	Wrong study design
Bhashyam, A. R.; Heng, M.; Harris, M. B.; Vrahas, M. S.; Weaver, M. J. (2018)	Self-Reported Marijuana Use Is Associated with Increased Use of Prescription Opioids Following Traumatic Musculoskeletal Injury	Wrong study design
Bisaga, A.; Sullivan, M. A.; Glass, A.; Mishlen, K.; Pavlicova, M.; Haney, M.; Raby, W. N.; Levin, F. R.; Carpenter, K. M.; Mariani, J. J.; Nunes, E. V. (2015)	The effects of dronabinol during detoxification and the initiation of treatment with extended release naltrexone	Wrong study design
Boehnke, K. F.; Litinas, E.; Clauw, D. J. (2016)	Medical Cannabis Use Is Associated with Decreased Opiate Medication Use in a Retrospective Cross-Sectional Survey of Patients with Chronic Pain	Wrong study design
Boehnke, K. F.; Scott, J. R.; Litinas, E.; Sisley, S.; Williams, D. A.; Clauw, D. J. (2019)	Pills to Pot: Observational Analyses of Cannabis Substitution Among Medical Cannabis Users With Chronic Pain	Wrong study design
Bulbul, A.; Mino, E. A.; Khorsand-Sahbaie, M.; Lentkowski, L. (2018)	Opioid dose reduction and pain control with medical cannabis	Unable to confirm details with authors
Chelliah, M. P.; Zinn, Z.; Khuu, P.; Teng, J. M. C. (2018)	Self-initiated use of topical cannabidiol oil for epidermolysis bullosa	Wrong study design
Chopda, G. R.; Parge, V.; Thakur, G. A.; Gatley, S. J.; Makriyannis, A.; Paronis, C. A. (2016)	Tolerance to the diuretic effects of cannabinoids and cross-tolerance to a kappa-opioid agonist in THC-treated mice	Wrong intervention
Cocchiara, E.; Spinella, A.; Magnani, L.; Lumetti, F.; Palermo, A.; Baiocchi, G.; Salvarani, C.; Giuggioli, D. (2019)	Cannabinoids in the treatment of pain related to systemic sclerosis skin ulcers: Our experience	Unable to confirm details with authors
Cooper, Z. D.; Comer, S. D.; Haney, M. (2017)	Opioid modulation of cannabis-induced analgesia and subjective effects in cannabis smokers	Abstract where full paper published
Cudmore, J.; Daeninck, P. J. (2015)	Use of medical cannabis to reduce pain and improve quality of life in cancer patients	Full text unavailable
Cuñetti, L.; Manzo, L.; Peyraube, R.; Arnaiz, J.; Curi, L.; Orihuela, S. (2018)	Chronic Pain Treatment With Cannabidiol in Kidney Transplant Patients in Uruguay	No outcome measure of interest
Curtis, S. A.; Lew, D.; Spodick, J.; Hendrickson, J. E.; Minniti, C. P.; Roberts, J. D. (2020)	Medical marijuana certification for patients with sickle cell disease: A report of a single center experience	Wrong study design
Darnall, B. D.; Humphreys, K. N. (2018)	An experimental method for assessing whether marijuana use reduces opioid use in patients with chronic pain	Wrong study design
Datta, U.; Kelley, L. K.; Middleton, J. W.; Gilpin, N. W. (2020)	Positive allosteric modulation of the cannabinoid type-1 receptor (CB1R) in periaqueductal gray (PAG) antagonizes anti-nociceptive and cellular effects of a mu-opioid receptor agonist in morphine-withdrawn rats	Wrong intervention
Davies, E.; Boge, J.; Salas, M.	Dronabinol for the treatment of veterans with chronic pain: A retrospective case series	Unable to confirm details with authors
de Almeida, A. S.; Rigo, F. K.; De Pra, S. D. T.; Milioli, A. M.; Dalenogare, D. P.; Pereira, G. C.; Ritter, C. S.; Peres, D. S.; Antoniazzi, C. T. D.; Stein, C.; Moresco, R. N.; Oliveira, S. M.; Trevisan, G. (2019)	Characterization of Cancer-Induced Nociception in a Murine Model of Breast Carcinoma	Wrong intervention
De Aquino, J. P.; Sofuoglu, M.; Stefanovics, E. A.; Rosenheck, R. A. (2020)	Impact of cannabis on non-medical opioid use and symptoms of posttraumatic stress disorder: a nationwide longitudinal VA study	Wrong study design
Ding, H.; Kiguchi, N.; Kishioka, S.; Ko, M. C (2016)	Comparison of heroin-and delta⁹-tetrahydrocannabinol-induced antinociception and physical dependence in monkeys	Full text unavailable
Dusi, V.; Attili, S. V. S.; Singaraju, M. (2019)	Observational study on role of crude cannabis in pain control and quality of life in terminally ill cancer patients: An Indian perspective	Unable to confirm details with authors
Dvoracsko, S.; Keresztes, A.; Mollica, A.; Stefanucci, A.; Macedonio, G.; Pieretti, S.; Zador, F.; Walter, F. R.; Deli, M. A.; Kekesi, G.; Banki, L.; Tuboly, G.; Horvath, G.; Tomboly, C. (2019)	Preparation of bivalent agonists for targeting the mu opioid and cannabinoid receptors	Wrong intervention
Gallo, E.; Maggini, V.; Comite, M.; Sofi, F.; Baccetti, S.; Vannacci, A.; Di Stefano, M.; Monechi, M. V.; Gori, L.; Rossi, E.; Firenzuoli, F.; Mediati, R. D.; Ballerini, G.	SENeCA Study: Observational study on the effect of medicinal cannabis on quality of life and nutritional outcomes	Full text unavailable
Gerak, L. R.; France, C. P. (2016)	Combined treatment with morphine and DELTA⁹-tetrahydrocannabinol in rhesus monkeys: Antinociceptive tolerance and withdrawal	No outcome measure of interest

Gerak, L. R.; Zanettini, C.; Koek, W.; France, C. P. (2015)	Cross-tolerance to cannabinoids in morphine-tolerant rhesus monkeys	Wrong intervention
Gilpin, N. W.; Datta, U.; Avegno, E. M.; Lobell, T. D.; Itoga, C. A.; Weera, M. M.; Edwards, S.; Middleton, J. W. (2019)	Periaqueductal gray (PAG) plasticity in hyperalgesic rats dependent on alcohol or morphine	Unable to confirm details with authors
Giorgi, V.; Bongiovanni, S.; Atzeni, F.; Marotto, D.; Salaffi, F.; Sarzi-Puttini, P. (2020)	Adding medical cannabis to standard analgesic treatment for fibromyalgia: a prospective observational study	Unable to confirm details with authors
Giorno, T. B. S.; Moreira, I. G.; Rezende, C. D. M.; Fernandes, P. D. (2018)	A new N-alkanoyl-5-hydroxytriptamide produces central antinociception and ameliorates thermal hyperalge sia	Unable to confirm details with authors
Godoi, M. M.; Junior, H. Z.; da Cunha, J. M.; Zanoveli, J. M. (2020)	Mu-opioid and CB1 cannabinoid receptors of the dorsal periaqueductal gray interplay in the regulation of fear response, but not antinociception	Wrong intervention
Gonzalez-Rodriguez, S.; Poras, H.; Menendez, L.; Lastra, A.; Ouimet, T.; Fournie-Zaluski, M. C.; Roques, B. P.; Baamonde, A. (2017)	Synergistic combinations of the dual enkephalinase inhibitor PL265 given orally with various analgesic compounds acting on different targets, in a murine model of cancer-induced bone pain	Wrong intervention
Grenald, S.; Guan, Y.; Raja, S. (2018)	Peripheral cannabinoid and mu opioid receptor synergistic inhibition of neuropathic pain	Unable to confirm details with authors
Grenald, S.; Wang, Y.; Young, M.; Stark, J.; Hu, J.; Vanderah, T. (2015)	Synergistic drug interaction between morphine and a cannabinoid receptor 2 agonist in a model of neuropathic pain	Unable to confirm details with authors
Harris, H. M.; Rousseau, M. A.; Wanas, A. S.; Radwan, M. M.; Caldwell, S.; Sufka, K. J.; Elsohly, M. A. (2019)	Role of Cannabinoids and Terpenes in Cannabis-Mediated Analgesia in Rats	Wrong intervention
Hauser, W.; Welsch, P.; Klose, P.; Radbruch, L.; Fitzcharles, M. A. (2019)	Efficacy, tolerability and safety of cannabis-based medicines for cancer pain: A systematic review with meta-analysis of randomised controlled trials	Wrong study design
Hicks, K.; Snyder, C. (2018)	Impact of high-dose cannabis use in patients with advanced pancreatic cancer undergoing treatment in a phase i clinical trial: Lessons learned and impact on future clinical research design	Unable to confirm details with authors
Higgins, P.; Ginsburg, D.; Gilder, K.; Walsh, B.; English, B.; Turner, S.; Klassen, P.; Hanauer, S.; Barish, C.; Yacyshyn, B. (2019)	Safety and efficacy of olorinab, a peripherally restricted, highly-selective, cannabinoid receptor 2 agonist in a phase 2A study in chronic abdominal pain associated with Crohn's disease	Wrong intervention
Huang, I. C.; Buckley, M.; Zhenghong, L.; Ehrhardt, M.; Alberts, N.; Brinkman, T.; Allen, J.; Krull, K.; Klosky, J.; Greene, W.; Srivastava, D. K.; Robison, L.; Hudson, M.; Anghelescu, D. (2018)	Persistent bodily pain and use of opioids and marijuana: A longitudinal study among adult survivors of childhood cancer in the st. jude lifetime cohort (SJLIFE)	Wrong study design
Hutchison, K. E.; Hagerty, S. L.; Galinkin, J.; Bryan, A. D.; Bidwell, L. C.(2019)	Cannabinoids, Pain, and Opioid Use Reduction: The Importance of Distilling and Disseminating Existing Data	Wrong study design
Ishida, J. H.; Wong, P. O.; Cohen, B. E.; Vali, M.; Steigerwald, S.; Keyhani, S. (2019)	Substitution of marijuana for opioids in a national survey of US adults	Wrong study design
Iyer, V.; Slivicki, R. A.; Thomaz, A. C.; Crystal, J. D.; Mackie, K.; Hohmann, A. G. (2020)	The cannabinoid CB<inf>2</inf> receptor agonist LY2828360 synergizes with morphine to suppress neuropathic nociception and attenuates morphine reward and physical dependence	Duplicate
Jamal, N.; Korman, J.; Musing, M.; Malavade, A.; Coleman, B. L.; Siddiqui, N.; Friedman, Z. (2019)	Effects of pre-operative recreational smoked cannabis use on opioid consumption following inflammatory bowel disease surgery: A historical cohort study	Wrong study design
Jarmuz, A.; Zielinska, M.; Keasling, A.; Zjawiony, J.; Fichna, J. (2016)	Interactions between the endogenous opioid and cannabinoid systems in the gastrointestinal tract are crucial in the development of tolerance to opioids	Abstract where full paper published
Javid, H.; Rezayof, A.; Ghasemzadeh, Z.; Sardari, M. (2020)	The involvement of ventral hippocampal microglial cells, but not cannabinoid CB1 receptors, in morphine-induced analgesia in rats	Wrong study design
Jemos, C.; Villa, J.; Zuniga Guerrero, A. M.; Guardamagna, V. A.; Omodeo Sale, E. (2018)	The use of cannabis oil in oncological pain: Analysis of the outcomes in real practice at a cancer centre	Unable to confirm details with authors
Jennings, J. M.; Angerame, M. R.; Eschen, C. L.; Phocas, A. J.; Dennis, D. A. (2019)	Cannabis Use Does Not Affect Outcomes After Total Knee Arthroplasty	Wrong study design
Jicha, C. J.; Lofwall, M. R.; Nuzzo, P. A.; Babalonis, S.; Elayi, S. C.; Walsh, S. L. (2015)	Safety of oral dronabinol during opioid withdrawal in humans	No outcome measure of interest
Kandasamy, R.; Dawson, C. T.; Hilgendorf, T. N.; Morgan, M. M. (2018)	Medication overuse headache following repeated morphine, but not [INCREMENT]9-tetrahydrocannabinol administration in the female rat	Wrong intervention
Koliani-Pace, J.; Gordon, S. R.; Gardner, T. B. (2017)	The effect of medical cannabis on pain and opioid use in chronic pancreatitis	Abstract where full paper published
Kreutzkamp, B.; Häuser, W. (2018)	Chronic pain: Only few patients on opioids benefit from additional (illegal) cannabis	Wrong study design
Levin, D. N.; Dulberg, Z.; Chan, A.; Hare, G.; Mazer, C.; Hong, A. (2016)	A randomized controlled trial of nabilone for the prevention of postoperative nausea and vomiting in elective surgery	Abstract where full paper published
Li, A. L.; Lin, X.; Dhopeshwarkar, A. S.; Thomaz, A. C.; Carey, L. M.; Liu, Y.; Nikas, S. P.; Makriyannis, A.; Mackie, K.; Hohmann, A. G. (2019)	Cannabinoid CB2 agonist AM1710 differentially suppresses distinct pathological pain states and attenuates morphine tolerance and withdrawal	Wrong intervention
Limbach, K. E.; Pommier, S. J.; Pommier, R. F.; Naik, A. M. (2019)	A prospective study of opioid use for postoperative pain management after breast surgery	Wrong study design
Lin, X.; Dhopeshwarkar, A. S.; Huibregtse, M.; MacKie, K.; Hohmann, A. G. (2018)	Slowly signaling G protein-biased CB<inf>2</inf> cannabinoid receptor agonist LY2828360 suppresses neuropathic pain with sustained efficacy and attenuates morphine tolerance and dependence	No outcome measure of interest
Lo Castro, F.; Baraldi, C.; Cainazzo, M. M.; Ferrari, A.; Pani, L.; Guerzoni, S. (2019)	Cannabis for the treatment of refractory headaches: A case-series of 18 patients	Unable to confirm details with authors

Lofwall, M. R.; Babalonis, S.; Nuzzo, P. A.; Elayi, S. C.; Walsh, S. L. (2016)	Opioid withdrawal suppression efficacy of oral dronabinol in opioid dependent humans	No outcome measure of interest
Maguire, D. R.; France, C. (2017)	Impact of delta-9-tetrahydrocannabinol on the reinforcing effects of remifentanil in rhesus monkeys responding under a food/drug choice procedure	Unable to confirm details with authors
Maguire, D. R.; France, C. P. (2018)	Reinforcing effects of opioid/cannabinoid mixtures in rhesus monkeys responding under a food/drug choice procedure	No outcome measure of interest
Maguire, D. R.; France, C. P.(2016)	Interactions between cannabinoid receptor agonists and mu opioid receptor agonists in rhesus monkeys discriminating fentanyl	No outcome measure of interest
Maguire, D.; Weed, P.; Gerak, L.; France, C. (2017)	Evaluation of abuse-and overdose-related effects of opioid/cannabinoid mixtures	Unable to confirm details with authors
Manini, A. F.; Yiannoulos, G.; Bergamaschi, M. M.; Hernandez, S.; Olmedo, R.; Barnes, A. J.; Winkel, G.; Sinha, R.; Jutras-Aswad, D.; Huestis, M. A.; Hurd, Y. L. (2015)	Safety and pharmacokinetics of oral Cannabidiol when administered concomitantly with intravenous Fentanyl in humans	No outcome measure of interest
Manz, J.; Hyakutake, M.; Kelly, E. (2020)	Calling for Openness to the Study of Cannabis Use in Chronic Pelvic Pain	Wrong study design
McVige, J.; Kaur, D.; Hart, P.; Lillis, M.; Mechtler, L.; Bargnes, V.; Shukri, S. (2019)	Medical cannabis in the treatment of post-traumatic concussion	Unable to confirm details with authors
Mechtler, L.; Bargnes, V.; Hart, P.; McVige, J.; Saikali, N. (2019)	Medical cannabis for chronic migraine: A retrospective review	Unable to confirm details with authors
Mechtler, L.; Hart, P.; Bargnes, V.; Saikali, N. (2019)	Medical cannabis treatment in patients with trigeminal neuralgia	Unable to confirm details with authors
Mechtler, L.; Ralyea, C.; Hart, P.; Bargnes, V. (2020)	Medical cannabis in the treatment of patients with trigeminal neuralgia: An ongoing retrospective study	Unable to confirm details with authors
Merlin, J. S.; Long, D.; Becker, W. C.; Cachay, E. R.; Christopolous, K. A.; Claborn, K. R.; Crane, H. M.; Edelman, E. J.; Lovejoy, T. I.; Mathews, W. C.; Morasco, B. J.; Napravnik, S.; O'Cleirigh, C.; Saag, M. S.; Starrels, J. L.; Gross, R.; Liebschutz, J. M. (2019)	Marijuana Use Is Not Associated with Changes in Opioid Prescriptions or Pain Severity among People Living with HIV and Chronic Pain	Wrong study design
Merlin, J. S.; Samet, J. H.; Cheng, D. M.; Lira, M. C.; Tsui, J. I.; Forman, L. S.; Colasanti, J.; Walley, A. Y.; Del Rio, C.; Liebschutz, J. M. (2019)	Marijuana Use and Its Associations with Pain, Opioid Dose, and HIV Viral Suppression among Persons Living with HIV on Chronic Opioid Therapy	Wrong study design
Miller, C.; Foster, C.; Mueller, S. (2015)	Dronabinol for acute pain management in burn patients that use marijuana	Unable to confirm details with authors
Minervini, V.; France, C. P. (2016)	Behavioral characterization of spiradoline and CP55,940 in rats: Potential of drug mixtures for treating pain	Abstract where full paper published
Molaei, M.; Fatahi, Z.; Zaringhalam, J.; Haghparast, A. (2016)	CB1 Cannabinoid Agonist (WIN55,212-2) Within the Basolateral Amygdala Induced Sensitization to Morphine and Increased the Level of mu-Opioid Receptor and c-fos in the Nucleus Accumbens	Wrong intervention
Mollica, A.; Pelliccia, S.; Famiglini, V.; Stefanucci, A.; Macedonio, G.; Chiavaroli, A.; Orlando, G.; Brunetti, L.; Ferrante, C.; Pieretti, S.; Novellino, E.; Benyhe, S.; Zador, F.; Erdei, A.; Szucs, E.; Samavati, R.; Dvrorasko, S.; Tomboly, C.; Ragno, R.; Patsilinakos, A.; Silvestri, R. (2017)	Exploring the first Rimonabant analog-opioid peptide hybrid compound, as bivalent ligand for CB1 and opioid receptors	Wrong study design
Mucke, M.; Phillips, T.; Radbruch, L.; Petzke, F.; Hauser, W. (2018)	Cannabis-based medicines for chronic neuropathic pain in adults	Wrong study design
Mupamombe, C. T.; Nathan, R. A.; Case, A. A.; Walter, M.; Hansen, E (2019)	Efficacy of medical cannabis for cancer-related pain in the elderly: A single-center retrospective analysis	Full text unavailable
Myers, B.; Geist, T.; Aladeen, T.; Westphal, E.; Hart, P.; Zelen, K.; Begley, A.; Rainka, M.; Florea, S.; Mechtler, L. (2020)	Medical cannabis in the treatment of Parkinson's disease	Unable to confirm details with authors
NCT	Prospective Blinded Randomized Controlled Trial Evaluating the Outcomes of Cannabinoid (CBD) Roll-on Topical Stick in Primary Total Knee Arthroplasty	Unable to confirm details with authors
NCT	Investigation of Cannabis for Pain and Inflammation in Lung Cancer	Clinical trial withdrawn
NCT	Inhaled Cannabis Versus Fentanyl Buccal Tablets for Management of Breakthrough Pain in Cancer Patients	Unable to confirm details with authors
NCT	Cannabis Versus Oxycodone for Pain Relief	Wrong intervention
Ngan, T. Y. T.; Litt, M.; Eguzo, K.; Thiel, J. A. (2019)	Patient Outcomes Following Initiation of Medical Cannabis in Women with Chronic Pelvic Pain	Unable to confirm details with authors
Nguyen, J. D.; Grant, Y.; Creehan, K. M.; Hwang, C. S.; Vandewater, S. A.; Janda, K. D.; Cole, M.; Taffe, M. A. (2019)	DELTA⁹-tetrahydrocannabinol attenuates oxycodone self-administration under extended access conditions	Duplicate
Nilges, M. R.; Bondy, Z.; Grace, J. A.; Winsauer, P. (2018)	Cannabinoid type-1 receptors can mediate the antinociceptive effects of heroin in nonhuman primates	Abstract where full paper published
Nilges, M. R.; Winsauer, P. (2017)	Persistent potentiation of the analgesic effects of opioids by delta-9Tetrahydrocannabinol (THC) in nonhuman primates	Abstract where full paper published
O'Connell, M.; Sandgren, M.; Frantzen, L.; Bower, E.; Erickson, B. (2019)	Medical Cannabis: Effects on Opioid and Benzodiazepine Requirements for Pain Control	No cannabis dose reported
Okusanya, B. O.; Asaolu, I. O.; Ehiri, J. E.; Kimaru, L. J.; Okechukwu, A.; Rosales, C. (2020)	Medical cannabis for the reduction of opioid dosage in the treatment of non-cancer chronic pain: A systematic review	Wrong study design
Ozdemir, E. (2020)	The role of the cannabinoid system in opioid analgesia and tolerance	Wrong study design

Pawasarat, I. M.; Schultz, E. M.; Frisby, J. C.; Mehta, S.; Angelo, M. A.; Hardy, S. S.; Kim, T. W. B (2020)	The Efficacy of Medical Marijuana in the Treatment of Cancer-Related Pain	Wrong intervention
Pearson, B. (2018)	Cannabis use as an alternative for opioids, antipsychotics and antidepressants among geriatric patients in a long-term care setting: A case series	Full text unavailable
Perez, J.; Olivier, S.; Rampakakis, E.; Borod, M.; Shir, Y. (2016)	The McGill university health centre cancer pain clinic: A retrospective analysis of an interdisciplinary approach to cancer pain management	Wrong study design
Pirasteh, A.; Molaee, M.; Haghparast, A. (2015)	Effect of intra-BLA administration of CB1 cannabinoid receptor agonist on sensitization to antinociceptive effect of morphine and expression of m-opioid receptor in the nucleus accumbens	Abstract where full paper published
Poli, P.; Crestani, F.; Salvadori, C.; Valenti, I.; Sannino, C. (2018)	Medical cannabis in patients with chronic pain: Effect on pain relief, pain disability, and psychological aspects. A prospective non randomized single arm clinical trial	Wrong intervention
Poli, P.; Salvadori, C.; Sannino, C. (2018)	Effects of cannabis based drugs on chronic neuropathic pain: Comparison between italian and dutch medical cannabis variety	Unable to confirm details with authors
Portman, D.; Donovan, K. A.; Bobonis, M. (2020)	Medical Cannabis as an Effective Treatment for Refractory Symptoms of Paraneoplastic Stiff Person Syndrome	Wrong study design
Pritchard, E. R.; Dayer, L.; Belz, J.; Forseth, B.; Harrington, S. E.; Painter, J. T. (2020)	Effect of cannabis on opioid use in patients with cancer receiving palliative care	Wrong intervention
Renard, O.; Chvetzoff, G.; Corbin, S.; Drouet, Y.; Lasset, C. (2019)	Cannabis and analgesic management: What are the consequences for the prescription of strong opioids? Observational study at the Léon-Bérard center in a lung cancer patients cohort	Wrong study design
Robinson, D.; Garti, A.; Yassin, M. (2016)	Cannabis treatment of diabetic neuropathy: Treatment effect and change in health over a 6 month period	Unable to confirm details with authors
Rogers, A. H.; Bakhshaie, J.; Buckner, J. D.; Orr, M. F.; Paulus, D. J.; Ditre, J. W.; Zvolensky, M. J. (2019)	Opioid and Cannabis Co-Use among Adults with Chronic Pain: Relations to Substance Misuse, Mental Health, and Pain Experience	Wrong study design
Ron, A.; Abuhasira, R.; Novack, V. (2019)	Establishment of a specialized geriatric clinic providing medical cannabis	Unable to confirm details with authors
Runner, R. P.; Luu, A. N.; Nassif, N. A.; Scudday, T. S.; Patel, J. J.; Barnett, S. L.; Gorab, R. S. (2020)	Use of Tetrahydrocannabinol and Cannabidiol Products in the Perioperative Period Around Primary Unilateral Total Hip and Knee Arthroplasty	Wrong study design
Salottolo, K.; Peck, L.; Tanner, A., II; Carrick, M. M.; Madayag, R.; McGuire, E.; Bar-Or, D. (2018)	The grass is not always greener: A multi-institutional pilot study of marijuana use and acute pain management following traumatic injury	Wrong study design
Serpell, M.; Ratcliffe, S.; Hovorka, J.; Schofield, M.; Taylor, L.; Lauder, H.; Ehler, E. (2014)	A double-blind, randomized, placebo-controlled, parallel group study of THC/CBD spray in peripheral neuropathic pain treatment	Unable to confirm details with authors
Shah, A.; Craner, J.; Cunningham, J. L. (2017)	Medical cannabis use among patients with chronic pain in an interdisciplinary pain rehabilitation program: Characterization and treatment outcomes	Wrong study design
Sing, D. C.; Tornetta, P.; Hansen, E. N. (2020)	The Role of Cannabis in Orthopedic Surgery	Wrong study design
Slivicki, R. A.; Iyer, V.; Mali, S. S.; Garai, S.; Thakur, G. A.; Crystal, J. D.; Hohmann, A. G. (2020)	Positive Allosteric Modulation of CB<inf>1</inf> Cannabinoid Receptor Signaling Enhances Morphine Antinociception and Attenuates Morphine Tolerance Without Enhancing Morphine- Induced Dependence or Reward	Wrong intervention
Smaga, S.; Gharib, A. (2017)	In adults with chronic low back pain, does the use of inhaled cannabis reduce overall opioid use?	Unable to confirm details with authors
Solomon, G. D.; Solomon, C. S. (2019)	Medical cannabis and chronic pain	Wrong study design
Sturgeon, J.; Hah, J.; Hilmoe, H.; Abrams, D.; Mackey, S.; Ware, M. (2018)	Clinical profiles of cannabis use in individuals with chronic pain: A CHOIR study	Wrong study design
Szymaszkiewicz, Agata; Świerczyński, Mikołaj; Talar, Marcin; Polepally, Prabhakar Reddy; Zjawiony, Jordan K.; Fichna, Jakub; Zielińska, Marta (2021)	Critical interactions between opioid and cannabinoid receptors during tolerance and physical dependence development to opioids in the murine gastrointestinal tract: proof of concept	No outcome measure of interest
Thompson, A. L.; Contreras, J. R.; Sorenson, J.; Lane, A. C.; Vanderah, T. (2020)	Role of opioids and cannabinoids in a murine model of bone fracture	Unable to confirm details with authors
Vigil, J. M.; Stith, S. S.; Adams, I. M.; Reeve, A. P. (2017)	Associations between medical cannabis and prescription opioid use in chronic pain patients: A preliminary cohort study	Wrong study design
Webster, E. M.; Yadav, G. S.; Gysler, S.; McNamara, B.; Black, J.; Tymon-Rosario, J.; Zeybek, B.; Han, C.; Arkfeld, C. K.; Andikyan, V.; Menderes, G.; Huang, G.; Azodi, M.; Silasi, D. A.; Santin, A. D.; Schwartz, P. E.; Ratner, E. S.; Altwerger, G. (2020)	Prescribed medical cannabis in women with gynecologic malignancies: A single-institution survey-based study	Wrong study design
Weed, P. F.; Gerak, L. R.; France, C. P. (2018)	Ventilatory-depressant effects of opioids alone and in combination with cannabinoids in rhesus monkeys	No outcome measure of interest
Withey, S. L.; Bergman, J. (2017)	Effects of cannabinoid receptor agonists on the antinociceptive effects of oxycodone in squirrel monkeys	Full text unavailable
Yu, Y.; Tsang, Q.; Lomax, A. E.; Vanner, S.; Reed, D. E. (2020)	Synergistic interactions of cannabinoids and opioids potentiate analgesia in mouse colonic nociception	Full text unavailable
Zador, F.; Wollemann, M. (2015)	Receptome: Interactions between three pain-related receptors or the "triumvirate" of cannabinoid, opioid and TRPV1 receptors	Wrong study design
Zylla, D. M.; Eklund, J.; Gilmore, G.; Gavenda, A.; Vazquez-Benitez, G.; Pawloski, P. A.; Arneson, T.; Birnbaum, A.; Dahmer, S.; Tracy, M.; Dudek, A. Z. (2019)	A randomized trial of medical cannabis (MC) in patients with advanced cancer (AC) to assess impact on opioid use and cancerrelated symptoms	Abstract where full paper published

Appendix 3. PRISMA diagram showing study identification

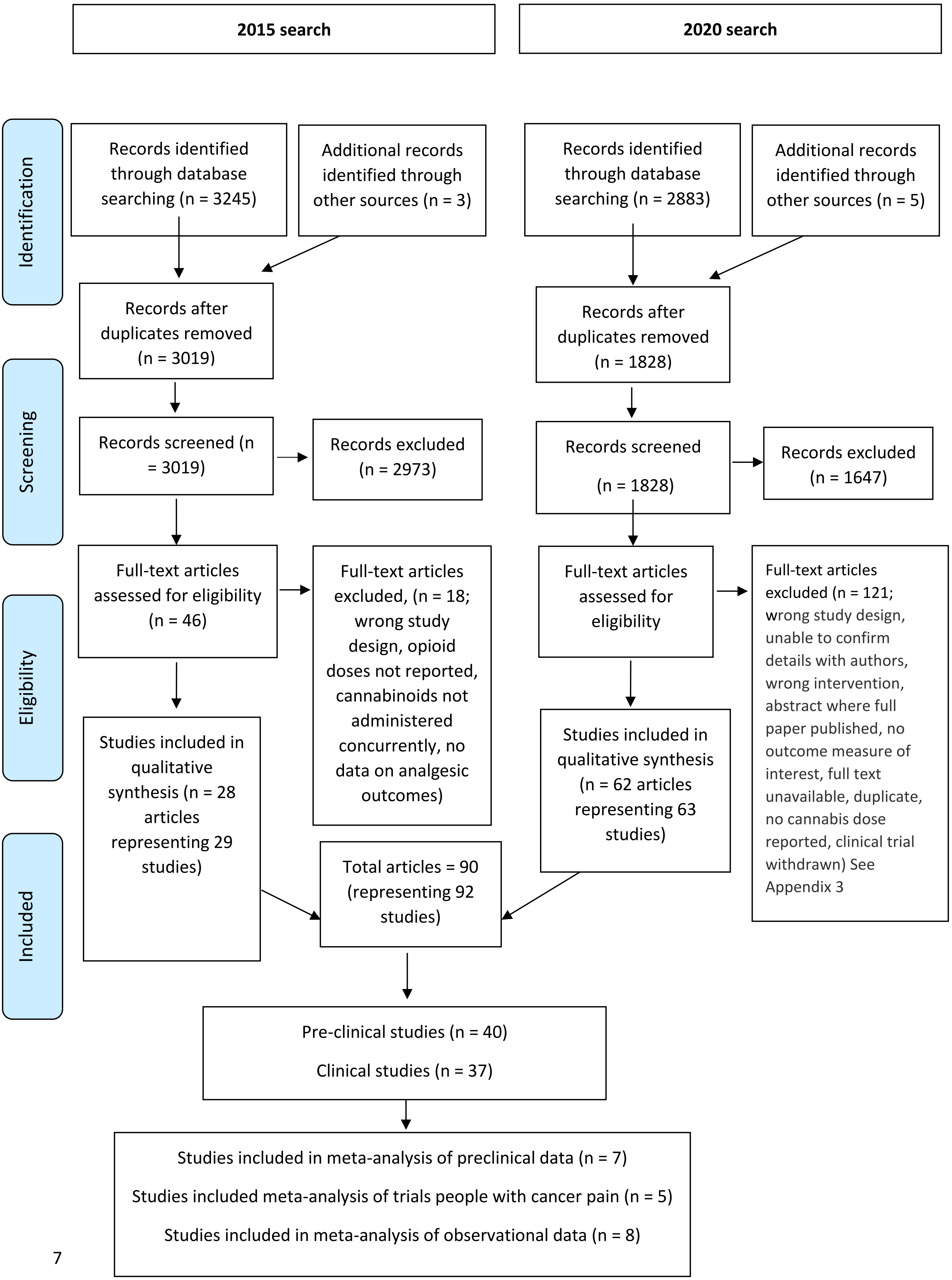

Appendix 4. Preclinical studies

Study	Species and pain model if relevant	Behavioural Measure/Pain Assay	Opioid administered	Cannabinoid administered	Equipotent opioid dose represented as ED50(95% CL) or ± SEM, unless measured otherwise specified		Potency ratio or evidence of synergism	Other notes
					Cannabinoid condition	Vehicle condition		
Alsalem 2019	Adult male Sprague-Dawley Rats. All received intraplantar CFA to left hindpaw (inflammation)	Von Frey filament test (mechanical allodynia).	Morphine I.P. (0.32, 1, 3.2 mg/kg)	CP55940 I.P. (0.032, 0.1, 0.32 mg/kg)	Not determined (dose-response used to determine minimum dose required to produce analgesia, MPE not determined)	Not determined (dose-response used to determine minimum dose required to produce analgesia, MPE not determined)	Potential synergy	Subtherapeutic doses of opioids used to assess changes in potency/efficacy of CP55940. Morphine (0.32mg/kg) increased efficacy and potency of CP55940 antinociception compared with vehicle, but tramadol had no effect at any dose tested. Values in red indicate doses of drug that produced antinociceptive effect when administered independently (single drug). In a separate group of rats, neither morphine nor tramadol in combination with CP55940 had an effect on intracranial self-stimulation, indicating these combinations are unlikely to increase the risk of abuse liability.
			Tramadol I.P. (1, 3.2, 10 mg/kg)	CP55940 I.P. (0.032, 0.1, 0.32 mg/kg)	Not determined (dose-response used to determine minimum dose required to produce analgesia, MPE not determined)	Not determined (dose-response used to determine minimum dose required to produce analgesia, MPE not determined)	No change in potency	
Alsalem 2020	Adult Male Sprague-Dawley rats (180-250g). CFA (inflammation) or saline (control) injected intraplantar to left hindpaw.	Von Frey filament test (mechanical nociception) and open field test (locomotion).	Morphine (0.32, 1, 3.2 mg/kg)	WIN55212 (0.1, 0.32, 1 mg/kg)	Not Determined	Not Determined	No change in potency	Subtherapeutic dose of opioids used to assess changes in potency/efficacy of WIN55212 and HU210 when given in combination compared with vehicle (no opioid). Values in red indicate doses of drug that produced antinociceptive effect when administered independently**. CFA:** Morphine and Tramadol displayed a trend to increase the efficacy of HU210 at its highest dose tested (1mg/kg), but this did not reach significance. **STZ:** morphine/HU210 displayed a trend for subadditivity. Subtherapeutic dose of Tramadol (1mg/kg) increased the efficacy of WIN55212 only at the highest dose tested (1mg/kg), suggesting potential synergy. **Open Field:** Neither opioid affected the locomotor deficits displayed by increasing doses of HU210 or WIN55121.
				HU210 (0.1, 0.32, 1 mg/kg)	Not Determined	Not Determined	No change in potency	
			Tramadol (1, 3.2, 10 mg/kg)	WIN55212 (0.1, 0.32, 1 mg/kg)	Not Determined	Not Determined	No change in potency	
				HU210 (0.1, 0.32, 1 mg/kg)	Not Determined	Not Determined	No change in potency	
	Adult Male Sprague-Dawley rats (180-250g). Streptosotocin (STZ; diabetic neuropathy) or vehicle (control) injected I.P.	Von Frey filament test (mechanical nociception) and open field test (locomotion).	Morphine (0.32, 1, 3.2 mg/kg)	WIN55212 (0.1, 0.32, 1 mg/kg)	Not Determined	Not Determined	No change in potency	
				HU210 (0.1, 0.32, 1 mg/kg)	Not Determined	Not Determined	No, interaction possibly subadditive.	
			Tramadol (1, 3.2, 10 mg/kg)	WIN55212 (0.1, 0.32, 1 mg/kg)	Not Determined	Not Determined	Potential synergy (see comment)	
				HU210 (0.1, 0.32, 1 mg/kg)	Not Determined	Not Determined	No change in potency	
Altun 2015	Adult Male Wistar Rats Naïve, Control or Morphine Dependent. No injury model.	Tail-flick and hot-plate tests (thermal nociception)	Morphine S.C. (5mg/kg)	ACEA I.P. (5mg/kg), JTE907 (5 mg/kg), AM251 (1 mg/kg), JWH-015(5 mg/kg)	Not Determined, single doses used in all conditions	Not Determined, single doses used in all conditions	Interaction unclear, potentially subadditive (see comment)	Single doses of opioid and cannabinoids administered either alone or in combination. Both cannabinoids in combination with morphine increased antinociception compared with morphine alone on tail-flick and hot-plate tests. Interaction unclear since both cannabinoids displayed strong antinociceptive properties, similar to morphine, when delivered alone. CB1R and CB2R antagonists reduced morphine antinociception on both tail-flick and hotplate tests. Ina assays of morphine tolerance, both cannabinoids enhanced morphine tolerance whilst both antagonists reduced morphine tolerance.

Auh 2016	Adult Male Sprague-Dawley Rats. CFA injected intraplantar into right hindpaw	Randall–Selitto Pressure Test (Mechanical nociception)	DAMGO, Intraplantar (0.001-1mg)	ACPA, Intraplantar (0.001 - 2mg). ED50 calculated as 111.6 (± 2.18) µg	128.4 ± 2.28 µg (ED50 of ACPA/DAMGO in combination)	57.4 ± 2.49 µg (ED50 of DAMGO alone) and 111.6 ± 2.18 µg for ACPA alone	ED50 increased above predicted additive effect. Drug interaction is subadditive.	1:1.9 fixed ratio combinations relative to potency (ED50) of DAMGO: ACPA. The experimentally derived ED50 was compared with the theoretical additive ED50 using isobolographic analysis.
Chen 2019	Male Swiss-Webster Mice	Formalin pain assay (5% formalin solution injected S.C. to dorsal side of left hindpaw). Measured late phase licking (20-35 mins post formalin injection).	Morphine, S.C. (0.5-10 mg/kg)	WIN55212-2; S.C. (0.5-5 mg/kg)	0.13 (0.11-0.18) mg/kg	0.43 (0.32-071) mg/kg	ED50 was below predicted additive value. Interaction is synergistic.	Morphine & WIN55212-2 produced dose-dependent antinociception when administered alone. 1:1 fixed ratio combinations and experimentally derived ED50 for this combination was compared with the theoretical additive ED50 using isobolographic analysis. Synergy was shown to be mediated by CB1 receptors.
				GP1a (CB2R agonist & FAAH inhibitor); S.C. (1, 5, 10 mg/kg)	Not determined	Not reported	Drug interaction was subadditive	GP1a produced analgesia in a non-dose related manner. Using dose equivalent analysis, the combinations of GP1a and morphine produced analgesia in a subadditive manner.
	Male Sprague-Dawley Rats. Carrageenan inflammatory pain assay; Carrageenan (2%) injected intraplantar to left hindpaw.	Thermal nociception and oedema measured with beam of radiant heat (Hargreaves test) and plethysometer (respectively).	Morphine, S.C. (3 mg/kg)	WIN55212-2; I.P. (1-5 mg/kg)	Not determined	3.37 (2.45-4.44) mg/kg	No evidence of synergy	Morphine produced analgesia in a dose-dependent manner but had no effect on oedema. Neither WIN55212-2 nor GP1a had analgesic activity and had no effect on oedema.
				inhibitor); I.P. (5 mg/kg) GP1a (CB2R agonist & FAAH	Not determined	Not reported	Potential evidence of synergy (see notes)	Single dose of morphine combined with 3 doses of WIN55212-2 had no effect on morphine analgesia and did not improve oedema. Single dose combination of morphine/GP1a increased morphine analgesia but did not improve oedema.
Cichewicz 1999	Male ICR mice (no injury model)	Tail-flick test	morphine p.o.	delta-9-THC (20 mg/kg p.o.)	13.1 mg/kg (8.8, 19.5)	28.8 mg/kg (20.2, 41)	Potency ratio: 2.2	
			codeine p.o.	delta-9-THC (20 mg/kg p.o.)	5.9 mg/kg (1.4, 24.9)	139.9 mg/kg (75.2, 260.5)	Potency ratio: 25.8	
			oxymorphone p.o.	delta-9-THC (20 mg/kg p.o.)	0.5 mg/kg (0.3, 0.8)	2.6 mg/kg (1.7, 3.9)	Potency ratio: 5.0	
			hydromorphone p.o.	delta-9-THC (20 mg/kg p.o.)	0.4 mg/kg (0.2, 0.8)	5.6 mg/kg (3.2, 9.7)	Potency ratio: 12.6	
			methadone p.o.	delta-9-THC (20 mg/kg p.o.)	2.7 mg/kg (1.4, 5.2)	12.0 mg/kg (8.1, 17.9)	Potency ratio: 4.1	
			LAAM p.o.	delta-9-THC (20 mg/kg p.o.)	2.6 mg/kg (1.7, 3.9)	8.0 mg/kg (6.4, 10.1)	Potency ratio: 2.5	
			heroin p.o.	delta-9-THC (20 mg/kg p.o.)	5.4 mg/kg (1.7, 16.9)	26.1 mg/kg (12.7, 53.4)	Potency ratio: 4.1	
			meperidine p.o.	delta-9-THC (20 mg/kg p.o.)	11.1 mg/kg (4.2, 29.4)	86.2 mg/kg (52.8, 140.6)	Potency ratio: 8.9	
			fentanyl p.o.	delta-9-THC (20 mg/kg p.o.)	0.5 mg/kg (0.3, 0.8)	6.1 mg/kg (estimated from an extrapolated curve)	Not determined (50% MPE not seen)	
			pentazocine p.o.	delta-9-THC (20 mg/kg p.o.)	838.6 mg/kg (estimated from an extrapolated curve)	625.9 mg/kg (estimated from an extrapolated curve)	Not determined (50% MPE not seen)	
Cichewicz 2003	Male ICR mice	Tail-flick test	morphine p.o.	delta-9-THC (5–35 mg/kg and 1–27 mg/kg p.o.)	13.6 mg/kg ± 1.94	24.5 mg/kg ± 4.8	For each ratio tested, experimental values were less than the calculated additive values (synergism)	Fixed-ratio combinations of 9-THC with either morphine or codeine were tested for antinociceptive effects. The experimentally derived ED_{50} for each combination was compared with the theoretical additive ED_{50}, using an isobolographic analysis. All the fixed-ratio combinations tested produced greater
			codeine p.o.	delta-9-THC (5–30 mg/kg and 5–18 mg/kg p.o.)	20.1 mg/kg ± 3.0	78.2 mg/kg ±14.4	For each ratio tested, experimental values were less than the calculated additive values (synergism)	

								antinociception (synergy) than predicted from simple additivity
Cichewicz 2005	IAF hairless guinea pigs	Pin-prick test	fentanyl s.c.	delta-9-THC (50 mg/kg i.p.)	6.8 µg/kg (3.3, 14.2)	50.8 µg/kg (41.0, 63.0)	Greater than additive effect on antinociception. Potency ratio: 6.7 (1.8 to 17.0)	
			buprenorphine s.c.	delta-9-THC (50 mg/kg i.p.)	0.02 mg/kg (0.01, 0.05)	2.97 mg/kg (1.84, 4.81)	Greater than additive effect on antinociception. Enhanced potency in a non-parallel fashion	Not possible to compare the change in potency produced by delta-9-THC due to the non-parallel nature of the two dose–response curves for buprenorphine
			fentanyl t.d.	delta-9-THC (400 mg/kg t.d.)	2h: 254.9 µg/kg (202.90, 320.6)<br>4h: 176.3 µg/kg (144.3, 215.5)	2h: 928.6 µg/kg (599.5, 1438.3)<br>4h: 1067.0 µg/kg (840.4, 1356.1)	Potency ratio at 2h: 3.7<br>Potency ratio at 4h: 5.8	
			buprenorphine t.d.	delta-9-THC (400 mg/kg t.d.)	2h: 4.3 mg/kg (2.8, 6.8)<br>4h: 2.2 mg/kg (1.1, 4.6)	2h: 26.1 mg/kg (17.1, 39.9)<br>4h: 15.6 mg/kg (10.0, 24.5)	Potency ratio at 2h: 8.2<br>Potency ratio at 4h: 7.2	
Cox 2007	(Male Sprague–Dawley rats)	Paw pressure test	morphine i.p. (normal rats)	delta-9-THC (0.4 mg/kg ± 0.5 i.p.) (1:1 ratio THC:Morphine)	0.4 mg/kg ± 0.5	2.4 mg/kg (2.2, 2.8)	The combination of delta-9-THC and morphine showed synergism in both non-arthritic and arthritic rats	Results from normal rats included in the meta-analysis only
			morphine i.p. (arthritic rats)	delta-9-THC (0.6 mg/kg ± 0.55 i.p.) (1:1 ratio THC:Morphine)	0.6 mg/kg ± 0.55	2.2 mg/kg (1.9, 2.4).		
Finn 2004	Adult male Lister Hooded rats	Formalin evoked nociceptive behavior	morphine i.p.	delta-9-THC (1 mg/kg i.p.)	Not reported	Not reported	Not clearly synergistic. Potentially additive. Morphine (2 mg/kg) + delta-9-THC (1 mg/kg) had a significant effect on nociceptive behavior (compared to morphine alone but not delta-9-THC alone).	
Grenald 2017	Male Sprague-Dawley Rats. Acute post-operative pain (incision of left hindpaw, behavioural testing 24hr afterwards).	Hargreaves thermal radiant heat test	Morphine, I.P. (1, 3, 10 mg/kg)	JWH105, I.P. (1, 3, 10 mg/kg)	0.14 ± 0.36 mg/kg	3.56 ± 8.61 mg/kg	1:1 fixed ratio; leftward shift in dose-effect curve for both thermal and mechanical nociception. Drug interaction is synergistic	Isobolographic analysis confirmed synergy between morphine and JW105 in measures of nociception in acute inflammation, post-operative injury and neuropathic pain models but not in thermal nociception of naïve rodents. Study also showed combination treatment reduced drug preference in a conditioned place preference test (measure of addictive potential) and reduce impairments in GI tract transit, tested in rats and compared with morphine treatment alone.
		Von Frey test of mechanical nociception			0.11 ± 0.41 mg/kg	5.02 ± 26.86 mg/kg		
	Male Sprague-Dawley Rats. Spared nerve injury model of neuropathic pain	Hargreaves thermal radiant heat test			0.14 ± 0.36 mg/kg	3.94 ± 2.99 mg/kg	1:1 fixed ratio; leftward shift in dose-effect curve for both thermal and mechanical nociception. Drug interaction is synergistic	
		Von Frey test of mechanical nociception			0.11 ± 0.41 mg/kg	2.56 ± 4.68		
	ICR Mice	Tail flick test (thermal nociception)	Morphine, I.P. (1, 3, 10 mg/kg)	JWH015, I.P. (1, 10, 100 mg/kg)	39.2 ± 43.8 mg/kg	4.47 ± 2.66 mg/kg	1:3 fixed ratio combination of morphine:JWH105; Drug interaction is sub-additive.	
		Formalin flinch test (1.5% formalin injected into left hindpaw)	Morphine, I.P. (0.1, 0.3, 0.6, 1 mg/kg)	JWH015, I.P. (0.1, 1, 3, 10, 30, 100 mg/kg)	0.01 ± 0.02 mg/kg	0.38 ± 0.33 mg/kg	2:1 fixed ratio; Leftward shift in dose-effect curve. Drug interaction is synergistic.	
Iyer 2020	Adult Male C57BL/6J WT or CB2R KO mice. Paclitaxel model of chemotherapy-induced neuropathic pain	Von Frey test of mechanical nociception	Morphine, I.P. (0.42, 0.84, 1.67, 3.34, 6.68 mg/kg)	LY2828360 (CB2R agonist; 0.04, 0.09, 0.19, 0.38, 0.77 mg/kg)	1.069 (0.4489-1.343) mg/kg	6.682 (4.905-9.103) mg/kg	leftward shift in dose-effect curve and ED50 was lower than the theoretical additive value. Interaction is synergistic	1:1 fixed ratio combination. Also blocked morphine-induced condition place preference, in WT mice but not CB2R KO mice and attenuated naloxone-precipitated withdrawal in chronically treated mice. Combination

	Adult Male C57BL/6J WT Naïve mice	Hotplate test of thermal nociception	Morphine I.P. (1, 3, 10 mg/kg)	LY2828360 (3 mg/kg)	not determined	not determined	No evidence of synergy	treatment had no effect on morphine-induced impairments in colonic motility or morphine-tolerance.
Katsuyama 2013	Male mice of ddY strain	Capsaicin test	morphine (1.0 mg/kg s.c. and 100 pmol i.t.)	beta-caryophyllene (2.25 mg i.pl., CB2 receptor agonist)	ID_{50} 1.16 mg/kg (1.03, 1.32, systemic, s.c.) and 130.1 pmol (111.9, 156.4, spinal, i.t.)	ID_{50} 2.51 mg/kg (2.17, 2.97) (systemic, s.c.) and 193.7 pmol (165.7, 225.6, spinal, i.t.)	Morphine + beta-caryophyllene decreased licking/biting response p<0.05 compared to morphine + saline or beta-caryophyllene + jojoba wax.	Ineffective doses of beta-caryophyllene significantly enhanced morphine-induced antinociception
Kazantzis 2016	Adult Male C57BL/6 mice. Chronic constriction injury (neuropathic) or sham (control) of left common sciatic nerve.	Von Frey Filament Test (mechanical allodynia)	Morphine, S.C. (1.78-30 mg/kg)	WIN55212, S.C. (0.1-10 mg/kg) ED50 calculated as 2.1 (± 0.08) mg/kg	3.4 (± 0.1) mg/kg (ED50 of morphine/WIN55212 in combination)	10.1 (± 0.5) mg/kg (ED50 of morphine alone)	ED50 was below predicted additive value. Interaction is synergistic.	1:1 fixed ratio combinations of respective ED50(S) of morphine and WIN55212 (this equated to a 5.6:1 fixed weight ratio of morphine to WIN55212). The experimentally derived ED50 for this combination was compared with the theoretical additive ED50 using isobolographic analysis. When administered in combination, WIN55212 and morphine reduced both mechanical and cold allodynia in a synergistic manner but only had an additive effect on motor incoordination.
		Acetone test (cold allodynia)	Morphine, S.C. (1.78-30 mg/kg)	WIN55212, S.C. (0.1-10 mg/kg) ED50 calculated as 1.1 (± 0.07) mg/kg	2.4 (± 0.1) mg/kg (ED50 of morphine/WIN55212 in combination)	7.4 (± 0.7) mg/kg (ED50 of morphine alone)	ED50 was below predicted additive value. Interaction is synergistic.	
		Rotarod test of motor coordination	Morphine, S.C. (1.78-30 mg/kg)	WIN55212, S.C. (0.1-10 mg/kg) ED50 calculated as 1.2 (± 0.2) mg/kg	4.0 (± 0.1) mg/kg (ED50 of morphine/WIN55212 in combination)	6.1 (± 0.5) mg/kg (ED50 of morphine alone)	ED50 was not different from predicted additive value. Interaction is additive.	
Li 2008	Rhesus monkeys	Thermal antinociception	morphine s.c.	delta-9-THC (0.32 and 1.0 mg/kg s.c.)	ED_{80} 2.42 mg/kg	ED_{80} 6.36 mg/kg (3.81, 8.91)	Pre-treatment with delta-9-THC enhanced the antinociceptive effects of morphine	Morphine dose dependently increased the latency for monkeys to remove their tails from 50°C and 55°C water
Maguire 2013	Rhesus monkeys	Warm water tail withdrawal	morphine s.c.	CP 55,940 (0.01 mg/kg s.c.)<br>WIN 55,212 (0.32 mg/kg s.c.)	mean (n = 3) CP 55,940 0.23 mg/kg<br>WIN 55,212 0.24 mg/kg	1.26 mg/kg (mean, n = 3)	Pre-treatment with CP 55,940 resulted in a mean leftward shift to of –6.73-fold. Pre-treatment with WIN 55,212 resulted in mean leftward shift of –5.5-fold.	Antinociception from the combination appeared to be achieved without an increase in abuse liability
Maguire and France 2016	Adult Male Sprague Dawley rats (14 months old). No pain model (Naïve)	Tail flick test (thermal nociception)	Spiradoline, I.P. (KOR agonist)	CP55940, I.P	1:1 ratio: 3.65 (1.86-7.17) mg/kg; 3:1 ratio: 1.93 (1.14-3.26) mg/kg; 1:3 ratio: 5.98 (3.08-11.64)	9.56 ± (3.96-23.07) mg/kg	ED50s of each dose ratio combinations fell near the line of additivity and within the 95% CL. Interaction was additive.	Isobolographic analysis of 1:1, 1:3 and 3:1 ratio combination of CP55940/Spiradoline
Maguire and France 2018	Adult Male Sprague Dawley Rats (~ 4 months old)	Warm-water tail withdrawal procedure (50 degrees C) to measure thermal antinociception.	Etorphine I.P. (**3:1**, 0.001 - 0.02; **1:1** 0.001 - 0.013; **1:3**, 0-0.007 mg/kg)	CP55940 I.P. (0.04 - 0.58 mg/kg)	0.0034 [0.0028, 0.0042] mg/kg	0.0063 [0.0057, 0.0069] mg/kg	Potency ratio = 1.9 [1.5, 2.3]. Drug interaction is sub-additive with greater than predicted ED50	Isobolographic analysis was used to assed the nature of interaction between Etorphine/CP55940, Etorphine/THC, Morphine/CP55940 & Morphine/THC. Dose-effect calculated using 3:1, 1:1 or 1:3 equivalent ratio of opioid/cannabinoids. Reported ED50 values & potency ratio [95% confidence interval].
				CP55940 I.P. (0.07 - 1.16 mg/kg)	0.0021 [0.0015, 0.0029] mg/kg		Potency ratio = 3.4 [2.1, 4.6]. Drug interaction is additive	
				CP55940 I.P. (0.11 - 1.73 mg/kg)	0.0018 [0.0012, 0.0027] mg/kg		Potency ratio = 4.1 [2.5, 5.8]. Drug interaction is sub-additive with greater than predicted ED50	
			Etorphine I.P. (**3:1**, 0.001 - 0.02; **1:1**	THC I.P. (1.4 - 21.7 mg/kg)	0.0045 [0.0036, 0.0057] mg/kg	0.006 [0.0063, 0.0068] mg/kg	Potency ratio = 1.5 [1.2, 1.9]. Drug interaction is sub-additive with lower than	

			0.001 - 0.014; **1:3**, 0-0.005 mg/kg)				predicted maximal effect and greater than predicted ED50	
				THC I.P. (2.7 - 43.4 mg/kg)	0.0035 [0.0025, 0.0048] mg/kg		Potency ratio = 2.0 [1.5, 2.6]. Drug interaction is sub-additive with greater than predicted ED50.	
				THC I.P. (4.1 - 48.8 mg/kg)	0.003 [0.0026, 0.0034] mg/kg		Potency ratio = 2.2 [1.9, 2.6]. Drug interaction is sub-additive with lower than predicted maximal effect and greater than predicted ED50.	
			Morphine I.P. (**3:1**, 1.6 - 25.1; **1:1** 1 - 16.7; **1:3**, 0.5 - 8.4 mg/kg)	CPP55940 I.P. (0.02 - 0.33 mg/kg)	3.57 [2.49, 5.13] mg/kg	6.99 [5.23, 9.36] mg/kg	Potency ratio = 1.9 [1.2, 2.6]. Drug interaction is supra-additive with greater than predicted maximal effect.	
				CPP55940 I.P. (0.04 - 0.65 mg/kg)	2.23 [1.52, 3.28] mg/kg		Potency ratio = 2.7 [2.0, 3.5]. Drug interaction is synergistic with lower than predicted ED50.	
				CPP55940 I.P. (0.06 - 0.98 mg/kg)	1.63 [1.23, 2.16] mg/kg		Potency ratio = 3.7 [2.8, 4.7]. Drug interaction is synergistic with greater than predicted maximal effect.	
			Morphine I.P. (**3:1**, 1.3 - 20.9; **1:1** 0.9 - 14.0; **1:3**, 0.4 - 7.0 mg/kg)	THC I.P. (0.7 - 10.4) mg/kg	3.40 [2.14, 5.40] mg/kg	6.68 [4.89, 9.15] mg/kg	Potency ratio = 2.6 [1.0, 4.2] Drug interaction is additive	
				THC I.P. (1.3 - 20.7) mg/kg	2.38, [1.64, 3.46] mg/kg		Potency ratio = 3.5 [1.7, 5.3]. Drug interaction is additive	
				THC I.P. (1.9 - 31.0) mg/kg	0.87 [0.55, 1.39] mg/kg		Potency ratio = 9.8 [3.9, 15.7]. Drug interaction is additive.	
Minervini et al 2017	Adult Male Sprague Dawley rats (350 ± 5 g)	Warm-water tail withdrawal procedure (40, 50, 55 degrees C) to measure thermal nociception.	Spiradoline, I.P. (KOR agonist; 0.032-32 mg/kg)	CP55940, I.P. (0.0032-1.0 mg/kg)	1:1 ratio: 2.75 ± 0.29; 3:1 ratio: 6.59 ± 0.99; 1:3 ratio: 2.03 ± 0.36 mg/kg	7.74 mg/kg	ED50s of each fixed dose ratio combinations fell near the line of additivity. Drug interaction is additive.	Isobolographic analysis was used to assed the nature of interaction between CP55940 and Spiradoline at 1:1, 1:3 and 3:1 ratios. The interaction was additive for antinociception and additive or greater than additive for hypothermia and food-maintained responding.
Neelakantan 2015	Male, Swiss-Webster mice	Acetic acid-stimulated stretching	Morphine I.P. (0.32-10 mg/kg)	CBD I.P. (10-40 mg/kg)	Not reported	Not reported	Leftward shift in the observed versus expected dose-effects. Drug interaction is synergistic	Morphine produced dose-dependent antinociception/analgesia across all behavioural measures. CBD produced dose-dependent antinociception in acetic acid-stretching, partial dose-independent antinociception in acetic acid reduced feeding and had no effect on the hotplate test. Dose-effect of cotreatment calculated using 1:1 dose ratio of morphine/CBD based on the relative potencies of the two drugs in each behavioural measure.
		acetic acid decreased feeding	Morphine I.P. (0.1-0.32 mg/kg)	CBD I.P. (5-40 mg/kg)	Not reported	Not reported	Rightward and downward shift in the observed versus expected dose-effects. Drug interaction is sub-additive.	
	Male C57BL/6 mice	Hotplate Thermal antinociception	Morphine, I.P. (1.0-32 mg/kg)	CBD, (3.2-32 mg/kg)	Not reported	Not reported	Rightward shift in observed versus expected dose-effects. Drug interaction is sub-additive.	

Nguyen 2019	Adult Male and female Wistar Rats (44-48 weeks old)	Tail flick test (water bath at 52 °C)	Oxycodone, vapour inhalation 100 mg/mL oxycodone;<br><br>Oxycodone, S.C. (1, 2 mg/kg)	∆9-THC, vapour inhalation 50 ng/mL THC<br><br>D9-THC, I.P. (5, 10 mg/kg)	Not Determined	Not Determined	Interaction unclear, possibly additive or synergistic.	∆9-THC/oxycodone produced significantly greater antinociception than oxycodone alone both when injected or when inhaled. THC reduced oxycodone self-administration. It should be noted that rats used in antinociception experiments were not naive to the drugs. Rats that received vapour inhalation of opioids/cannabinoids had previously received vapour inhalation of heroin, oxycodone, methadone and THC in pilot experiments to determine exposure conditions. Rats that received injections of opioids/cannabinoids had previously received chronic vapour inhalation of THC.
Nilges 2020	Adult male rhesus monkeys. Acute thermal antinociception	Tail-flick (water bath, 52 degrees C)	Heroin, I.M. (0.032 - 0.32 mg/kg)	D9-THC 0.1 mg/kg<br>D9-THC 0.32 mg/kg<br>D9-THC 1 mg/kg<br>Cannabinol (CBN) 1 - 3.2 mg/kg<br>THC 0.32 + CBN 1 mg/kg	ED50 0.12 (0.06, 0.18) mg/kg.<br>ED50 0.07 (0.04, 0.10) mg/kg.<br>ED50 0.03 (0.026, 0.032) mg/kg.<br>Value not reported<br>Value not reported	ED50 0.1 (0.06, 0.14) mg/kg	No change in potency<br>No change in potency<br>3.6-fold increase in potency<br>No change in potency<br>No change in potency	∆9-THC potential abolished by CB1 antagonist rimonabant
Pugh 1996	Male ICR mice	Tail-flick test	morphine i.t.	delta-9-THC (6 µcg/mouse i.t., inactive analgesic dose)	0.01 mcg/mouse	0.318 mcg/mouse (2.825, 0.036)	Greater than additive effect observed, clear leftward shift of graph	
Reche 1996	Swiss albino mice	Tail-flick and hot plate test	morphine i.p.	delta-9-THC i.p.	NA- Only one dose of morphine (2 mg/kg i.p.) examined. Study measured change in ED_{50} of delta-9-THC.	NA- Only one dose of morphine (2 mg/kg i.p.) examined. Study measured change in ED_{50} of delta-9-THC.	NA	Morphine pre-administration shifted the dose–response curve for delta-9-THC to the left (a 2.5-fold shift for the tail-flick test and a 3-fold shift for the hot plate test). Analgesic effect blocked by SR-141,716 (cannabinoid antagonist) and naloxone
Rodríguez-Muñoz 201)	Male CD-1 mice. Acute thermal antinociception	Tail-flick (water bath (52 degrees C)	Morphine, i.c.v. (6 nmol)	CBD (cannabidiol) 10 nmol i.c.v	Not determined	Not determined	Not determined	Single doses: CBD increased effect of morphine by 30 % (estimated)
Sierra 2019	Adult male C57 mice. Chemotherapy (paclitaxel) induced peripheral neuropathic pain (CIPN). Mechanical allodynia	von Frey filaments	SNC80 0.1 mg/kg i.p. (delta-opioid agonist)	HU210 0.05 µg/kg i.p.	Not determined	Not determined	Potential synergy (see comment)	Combination of subthreshold doses of HU210 + SNC80 abolished CIPN induced mechanical allodynia
Smith 1998	Male ICR mice	Tail-flick and hot plate test	morphine s.c.<br>morphine p.o.	Tail-flick: delta-9-THC (4 mg/kg s.c.)<br>Tail-flick: delta-9-THC (20 mg/kg p.o.)	0.29 mg/kg (95% C.I. 0.04, 1.94)<br>2.8 mg/kg (2.0, 3.9)	2.81 mg/kg (2.24, 3.53)<br>31.7 mg/kg (22.4, 44.9)	Potency ratio: 8.5<br>Potency ratio: 7.6	Multiple conditions tested different combinations of s.c and p.o morphine. Only s.c. + s.c. and p.o. + p.o. for the tail-flick test are reported here. A paw withdrawal test was also conducted to demonstrate that enhancement of antinociception was not limited to the tail
Smith 2007	Male Sprague–Dawley rats	Paw withdrawal test	morphine s.c.	delta-9-THC (0.75 mg /kg i.p.)	ED_{80} morphine + delta-9-THC (0.75 mg/kg)	ED_{80} morphine alone (100 mg/kg)	Tolerance to morphine alone rapidly established; no loss of effect with low-dose combinations of morphine + delta-9-THC	A morphine pellet arm and delta-9-THC alone arm were not reported in this table due to difficulties in comparing doses between morphine formulations

Stachtari 2016	Adult male Wistar rats. Incisional pain model. Thermal hyperalgesia.	Hot plate (52 degrees C)	Tramadol 10 mg/kg i.p.	AM1241 1 mg/kg i.p.	Not determined	Not determined	Not determined	Single doses: AM1241 did not potentiate effect of tramadol
Tham 2005	Swiss male mice	Tail-flick and hot plate test	morphine s.c.	Tail-flick: CP55,940 (0.1–3 mg/kg s.c.)	3.31 mg/kg	11.3 mg/kg (9.6, 13.4)	Analyses showed greater than additive results (synergism)	
				Hot plate: CP55,940 (0.1–3 mg/kg s.c.)	7.54 mg/kg	29.4 mg/kg (27.3, 31.6)		
Wakley 2011	Male Sprague–Dawley rats	Paw pressure test	methadone i.p.	delta-9-THC (0.32–3.2 mg/kg i.p.)	Not reported (dose response curve shown)	ED_{50} in naïve rats, 1.27 mg/kg (95% C.I. 0.91, 1.91), ED_{50} in rats trained for discrimination, 3.49 mg/kg (95% C.I. 2.59, 5.31)	In opioid and delta-9-THC naïve rats, methadone 1.0 mg/kg significantly enhanced antinociceptive effect of delta-9-THC, however this was not observed in rats that were previously trained for drug discrimination tasks.	The rats trained for drug discrimination tasks had received repeated administration of opioids and cannabinoids over many months and may have been tolerant to drug effects at the doses administered
Welch 1992	Mice	Tail-flick and hot plate test	morphine i.t.	delta-9-THC (3.133 mcg/mouse)	0.15 mcg/mouse (0.11, 0.21)		yes	
				delta-9-THC (6.25 mcg/mouse)	0.05 mcg/mouse (0.03, 0.08)		yes	
				delta-8-THC (25 mcg/mouse)	0.05 mcg/mouse (0.02, 0.10)		yes	
				levonantradol (0.005mcg/mouse)	0.06 mcg/mouse (0.01, 0.24)		yes	
				CP-55,940 (0.01 mcg/mouse)	0.3 mcg/mouse (0.0, 0.10)	0.61 mcg/mouse (0.26, 1.44)	additive	
				CP-56,667 (0.5 mcg/mouse)	0.26 mcg/mouse (0.08, 0.82)		additive	
				11-hydroxy-delta 9-THC (3 mcg/mouse)	0.08 mcg/mouse (0.04, 0.19)		yes	
				dextronantradol (25 mcg/mouse)	0.51 mcg/mouse (0.36, 0.89)		No	
Williams 2006	Male ICR mice	Tail-flick test	Study 1: low-dose codeine (30 mg/kg) and morphine (20 mg/kg) and fully efficacious ED_{80}, codeine (100 mg/kg) and morphine (80 mg/kg).<br>Study 2: high-dose codeine (200 mg/kg) and morphine (100 mg/kg) (all p.o.)	delta-9-THC (20 mg/kg p.o., inactive analgesic dose)	ED_{80} codeine (30 mg/kg)<br>ED_{80} morphine (20 mg/kg)	ED_{80} codeine (200 mg/kg)<br>ED_{80} morphine (100 mg/kg)	A low dose of morphine (20 mg/kg) or codeine (30 mg/kg) with a single pre-treatment of an inactive dose of delta-9-THC produced the same efficacy (ED_{80}) as the high doses of each opioid alone. For codeine, delta-9-THC pre-treatment also increased the duration of action of the ED_{80} dose of codeine	Study 1: Pre-treatment with delta-9-THC did not enhance the fully efficacious dose of morphine but enhanced low-dose morphine and both doses of codeine, in addition to extending the time course.<br>Study 2: delta-9-THC restored analgesic efficacy after the time that the opioids had ceased being effective on their own (360 mins post dose for morphine and 120 min post dose for codeine)
Williams 2008	Diabetic and non-diabetic mice and rats	Tail-flick test	morphine s.c.	delta-9-THC (20 mg/kg p.o.) in non-diabetic mice	2.5 mg/kg (1.8, 3.4)	5.6 mg/kg (4.3, 7.2)	Yes	delta-9-THC significantly enhanced morphine-induced antinociception in both diabetic and non-diabetic mice
			morphine s.c.	delta-9-THC (20 mg/kg p.o.) in diabetic mice	0.84 mg/kg (0.79, 0.89)	6.1 mg/kg (5.2, 7.1)	Yes	
Wilson 2008	Male Sprague–Dawley rats	Hot plate test	morphine microinjections into PAG	HU-210 (5 µg)	Not reported (dose response curve shown)	Not reported (dose response curve shown)	No evidence of synergism. Morphine + HU-210 showed the greatest increase in hot plate latency (39.9 s ± 1.1 s), but was not significantly	HU-210 shown to prevent development of tolerance to morphine's antinociceptive effects. HU-210 pre-treatment enhanced subsequent morphine antinociception. Co-administration of HU-210 into the PAG attenuated morphine

							different from morphine alone (33.1 s ±4.0 s)	antinociception. The authors suggested that opioids and cannabinoids may have opposing actions within the PAG
Yesilurt 2003	Adult female Bulb-C mice	Tail-flick test	morphine topical	WIN 55, 212-2 (20 mg/ml, topical, mixed CB1-CB2 receptor agonist)	morphine (20 mg/ml) + WIN sustained analgesic effect of 50% analgesia over 4 hours	morphine (20 mg/ml) alone produced 18% analgesic effect, peak at 20 min then reduced	Antinociceptive effects were markedly potentiated (they peaked and were sustained at 30 min) compared to morphine response alone	
Yuill 2017	Adult Male C57BL6/J Mice (8-14 weeks).	Formalin test of inflammatory pain (2.5% intraplatar to single hindpaw)	Morphine, I.P. (0.01-10 mgkg)	JWH-133, I.P. (CB2R agonist; 0.01-10 mg/kg)	Acute (phase I formalin response) = 0.7236 ± 0,11 mg/kg; Inflammatory (phase II formalin response) = 0.6211 ± 0.063 mg/kg	Not reported	Antinociception on both acute and inflammatory formalin phases did not differ significantly from the predicted additive value. Drug interaction is additive.	JWH-133 produced dose-dependent anti-nociception during both acute and inflammatory phases of the formalin test (Male: Acute & inflammatory ED50s = 0.23 mg/kg; Females: Acute ED50 = 0.24 mg/kg, Chronic ED50 = 0.2mg/kg), without development of tolerance during chronic treatment (11days). JWH133 did not cause cross tolerance for morphine but morphine caused cross-tolerance for JWH-133 antinociception. Coadministration of JWH133 and morphine reduced morphine tolerance. 1:10 fixed dose ratio of JWH-133/Morphine and non-linear Isobolographic analysis were used to examine potential synergy on antinociception.
Zhang 2017	Adult Male Wistar Rats. Walker 256 tumour cell implantation in right hindpaw (cancer pain)	Paw withdrawal latency to radiant heat (Hargreaves test)	Morphine, S.C. (10 mg/kg)	AM1241, I.T. (0.07 ug)	Not Determined	Not Determined	No change in potency	Single doses administered twice daily for 8 days, antinociception tested daily. Drug interaction ascertained from data obtained on days 1-4. Antinociception in both assays was equivalent to morphine alone on days 1-4. AM1241 dose was subtherapeutic and reduced development of morphine tolerance, which was blocked by CB2R antagonist.
Zhang 2018	Male ICR Mice, Naïve.	Von Frey Filament and hot-Plate Tests (52 degrees C)	Morphine, S.C. (5 mg/kg)	AM1241, I.P. (0.3, 1, 3 mg/kg)	Not Determined	Not Determined	Potential synergy	AM1241 had no antinociceptive effects when administered alone. The highest dose of AM1241 (3 mg/kg) increased acute morphine antinociception on both von Frey and hotplate assays. Coadministration of AM1241/morphine reduced acute/chronic morphine tolerance and naloxone-precipitated jumping but had no effect on naloxone-precipitated diarrhea and did not alter spontaneous locomotor activity.
Zhang 2016	Adult Male Wistar Rats. Walker 256 tumour cell implantation in right hindpaw (cancer pain)	Von Frey filament and hotplate tests (52 degrees C)	Morphine, S.C. (10 mg/kg)	AM1241, I.T. (0.07 ug)	Not Determined	Not Determined	No change in potency	Single doses administered twice daily for 8 days, antinociception tested each day on both assays. Drug interaction ascertained from data obtained on day 1. Antinociception in both assays was equivalent to morphine alone. AM1241 dose was subtherapeutic and reduced development of morphine tolerance.

p.o. = oral administration, s.c. = subcutaneous, t.d. transdermal, i.p. = intraperitoneal, i.t. = intrathecal, MPE = maximum possible effect, ICR = Imprinting Control Region, pmol = picomol, ED = Effective dose, ID = Inhibitory Dose, delta-9-THC = delta-9-tetrahydrocannabinol, PAG = periaqueductal gray matter

Appendix 5. Proportion of participants in observational studies reporting cessation of opioid use (a: top panel), and reduction in opioid use (including those who ceased)(b: bottom panel)

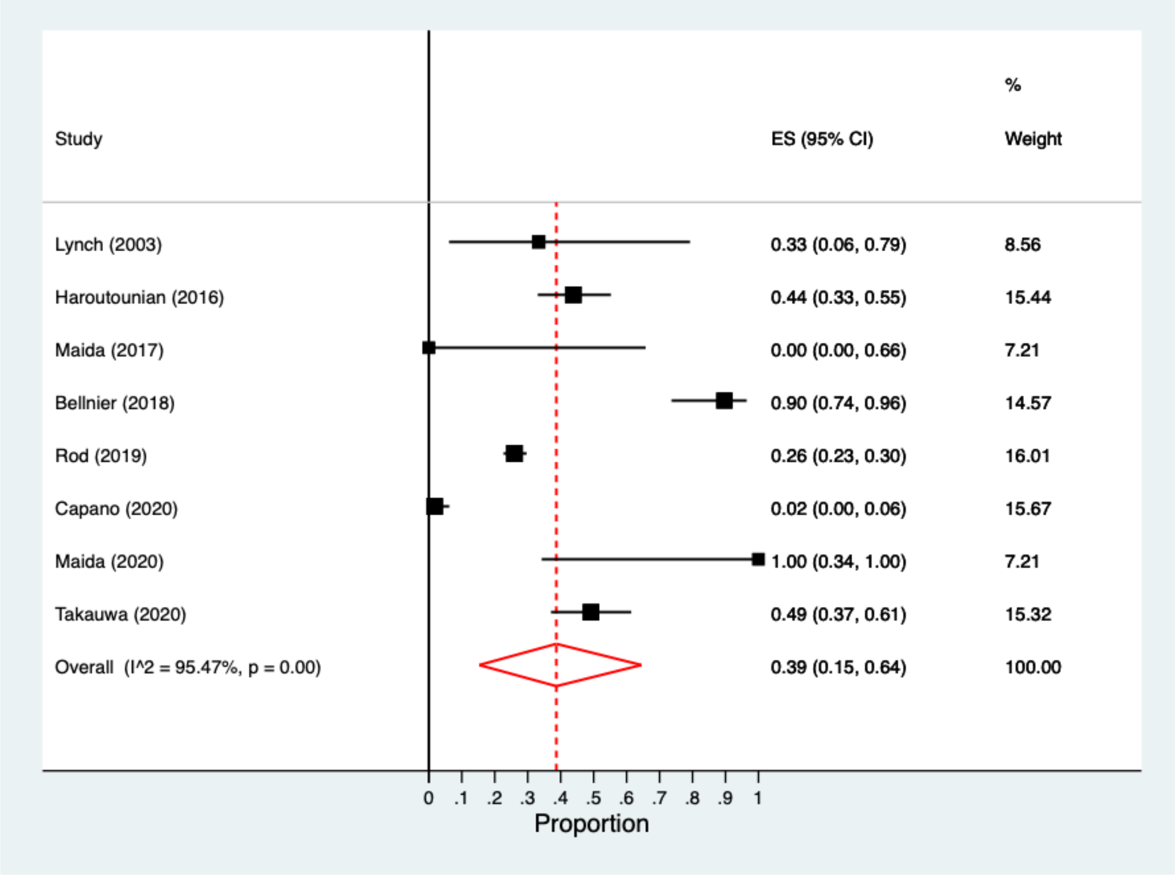

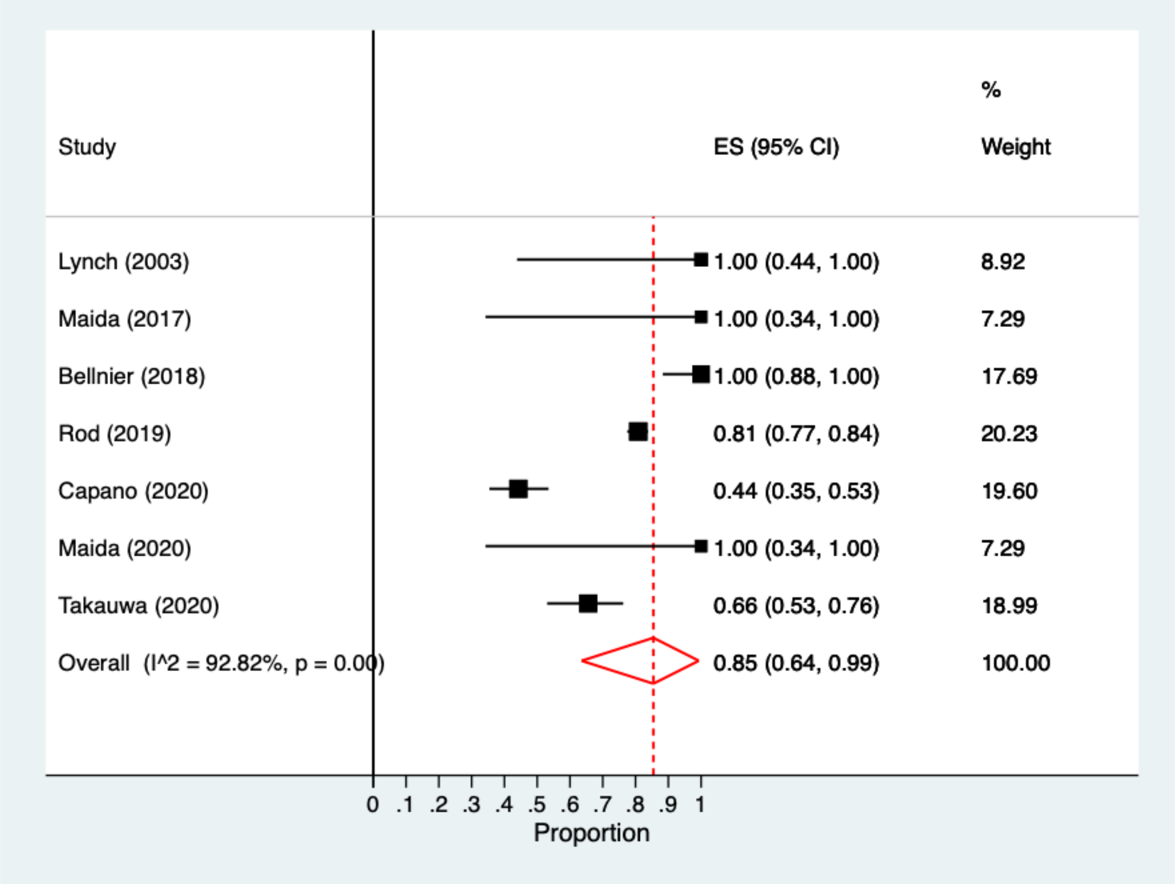

Appendix 6: Registered clinical trials, where results are not yet available (n=15)

Year	Title	Location	Trial status	Link
2018	A study that evaluates the effectiveness of oral medicinal cannabis for people with advanced cancer experiencing a range of symptoms	Australia	Recruiting	https://trialsearch.who.int/Trial2.aspx?TrialID=ACTRN12618001220257
2018	Inhaled Cannabis Versus Immediate -release Oral Opioids for Management of Breakthrough Pain in Cancer Patients	USA	Recruiting	https://clinicaltrials.gov/show/NCT03564548
2018	COPE: Cannabinoids to Obviate Pain Experiment After Knee Replacement	Canada	Not yet recruiting	https://clinicaltrials.gov/show/NCT03675971
2018	Cannabis Oil for Chronic Non-Cancer Pain Treatment (CONCEPT)	Canada	Unknown	https://clinicaltrials.gov/show/NCT03635593
2018	Nabilone Use For Acute Pain in Inflammatory Bowel Disease Patients	Canada	Not yet recruiting	https://clinicaltrials.gov/show/NCT03422861
2019	A study that evaluates the effectiveness of oral combined THC/CBD for people with advanced cancer experiencing a range of symptoms	Australia	Recruiting	https://anzctr.org.au/ACTRN12619000037101.aspx
2019	Interaction between opioids and cannabinoids in the treatment of fibromyalgia pain	Netherlands	Recruitment may be ongoing or finished	https://trialsearch.who.int/Trial2.aspx?TrialID=EUCTR2019-001861-33-NL
2019	Pain Response to Cannabidiol in Opioid-induced Hyperalgesia, Acute Nociceptive Pain and Allodynia By Using a Model Mimicking Acute Pain in Healthy Adults (CANAB II)	Switzerland	Completed (results not yet published)	https://clinicaltrials.gov/show/NCT04059978
2019	Cannabidiol, Morphine, Pain.	USA	Recruiting	https://clinicaltrials.gov/show/NCT04030442
2019	Cannabinoids vs. Placebo on Persistent Post-surgical Pain Following TKA: A Pilot RCT	Canada	Not yet recruiting	https://clinicaltrials.gov/show/NCT03825965
2019	Efficacy of dronabinol for acute pain management in adults with traumatic injury: Study protocol of a randomized controlled trial	USA	Terminated insufficient resources	https://clinicaltrials.gov/ct2/show/NCT03928015
2020	Do Discounted Vouchers for Medical Cannabis Reduce Opioid Use in Adults With Pain (ReLeaf-V)	USA	Recruiting	https://clinicaltrials.gov/show/NCT04495725
2020	Safety and Effects on Responses to Stress and Pain of Natural Medical Marijuana Product	USA	Not yet recruiting	https://clinicaltrials.gov/ct2/show/NCT04226690
2020	A Study to Assess the Effect of Cannabidiol Oil on Pain After Ureteroscopy for Kidney Stones	USA	Enrolling by invitation	https://clinicaltrials.gov/ct2/show/NCT04387617
2020	Prospective Blinded Randomized Controlled Trial Evaluating the Outcomes of Cannabinoid (CBD) Roll-on Topical Stick in Primary Total Knee Arthroplasty	USA	Enrolling by invitation	https://clinicaltrials.gov/show/NCT04585230
